# Supplementary figures and images for: Homeostatic bidirectional plasticity in upbound and downbound micromodules in a model of the olivocerebellar loop
Source: PLoS Comput Biol. 2025 Oct 21;21(10):e1013609. doi: 10.1371/journal.pcbi.1013609 (PMC12571319; doi:10.1371/journal.pcbi.1013609)

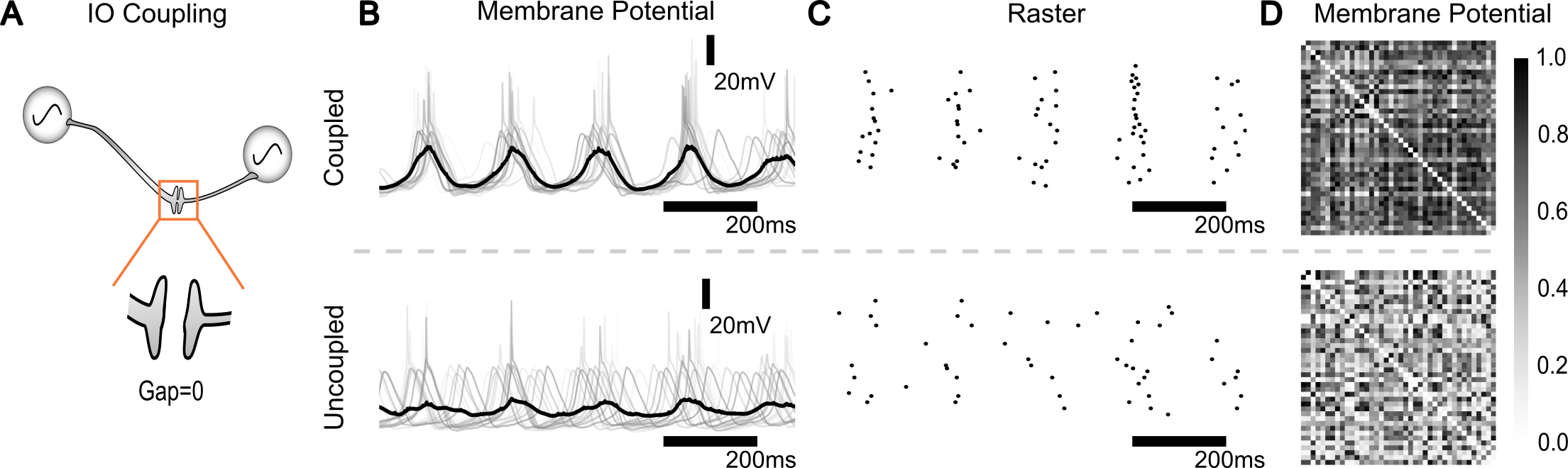

Supplement: S1 Fig — For the Downbound micromodule, we compare the IO membrane response (B), raster (C), and correlation matrix of membrane potentials (D) in coupled (top) and uncoupled networks (bottom). Synchrony directly impacts the correlations in weight changes expected at the PF-PC synapse. (TIF) [file pcbi.1013609.s001.tif]

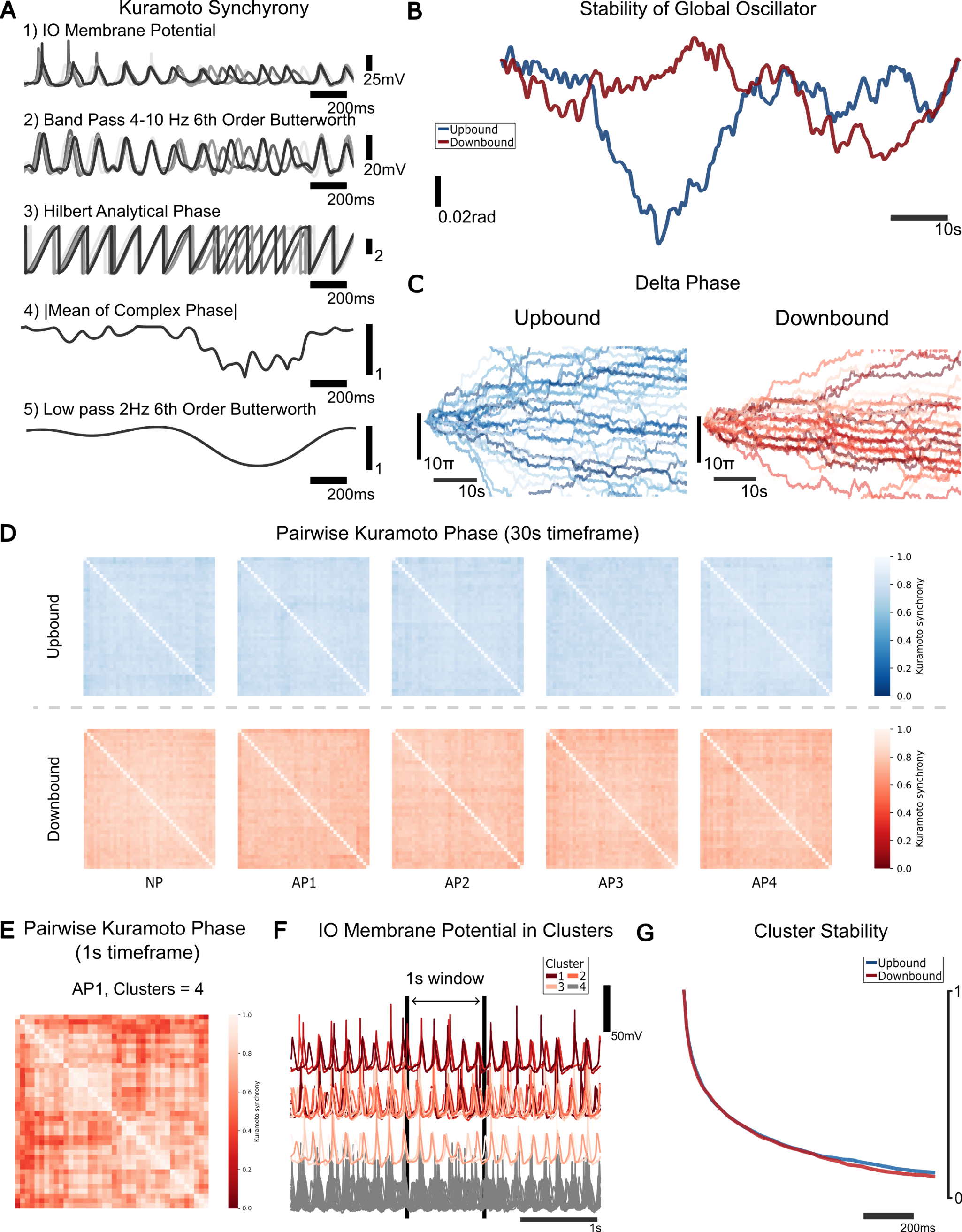

Supplement: S2 Fig — In a single micromodule we observe a few clusters in the short time scale whose stability fades exponentially. (A) Kuramoto synchrony metric for STOs is derived from IO Vm in 5 transformation steps. (B) Stability of global oscillator (i.e., usability as a low dimensional global clock): global phase (mean Hilbert phase of all IO cells) from start to end of an 80 second period. The deviation from horizontal indicates phase drifts across phase in radians. Both Upbound and Downbound IO have small drifts and maintain a robust global phase, equating with a single synchronized cluster. (C) Delta phase plot. Difference of phase of each IO neuron to the global phase over time. Plotted modulo 32pi radians to preserve space. Phase-locked oscillators are clearly visible as overlapping lines, while lag/leap events are shown as vertical jumps. Comparing long (D, 30s) and short term (E, 1s) clusterization with the pairwise kuramoto phase reveals a few clusters but a single global oscillator in the 30s frame. (F) Voltage trace of the clusters found in E. The black bars indicate the 1s used for clustering. (G) Cluster stability of 1s clusters. In intervals of 10ms, 1s clusters were found with HDBSCAN [121] and compared with each other via NMI (normalized mutual information). At a distance of 1s, clusters are barely related. (TIF) [file pcbi.1013609.s002.tif]

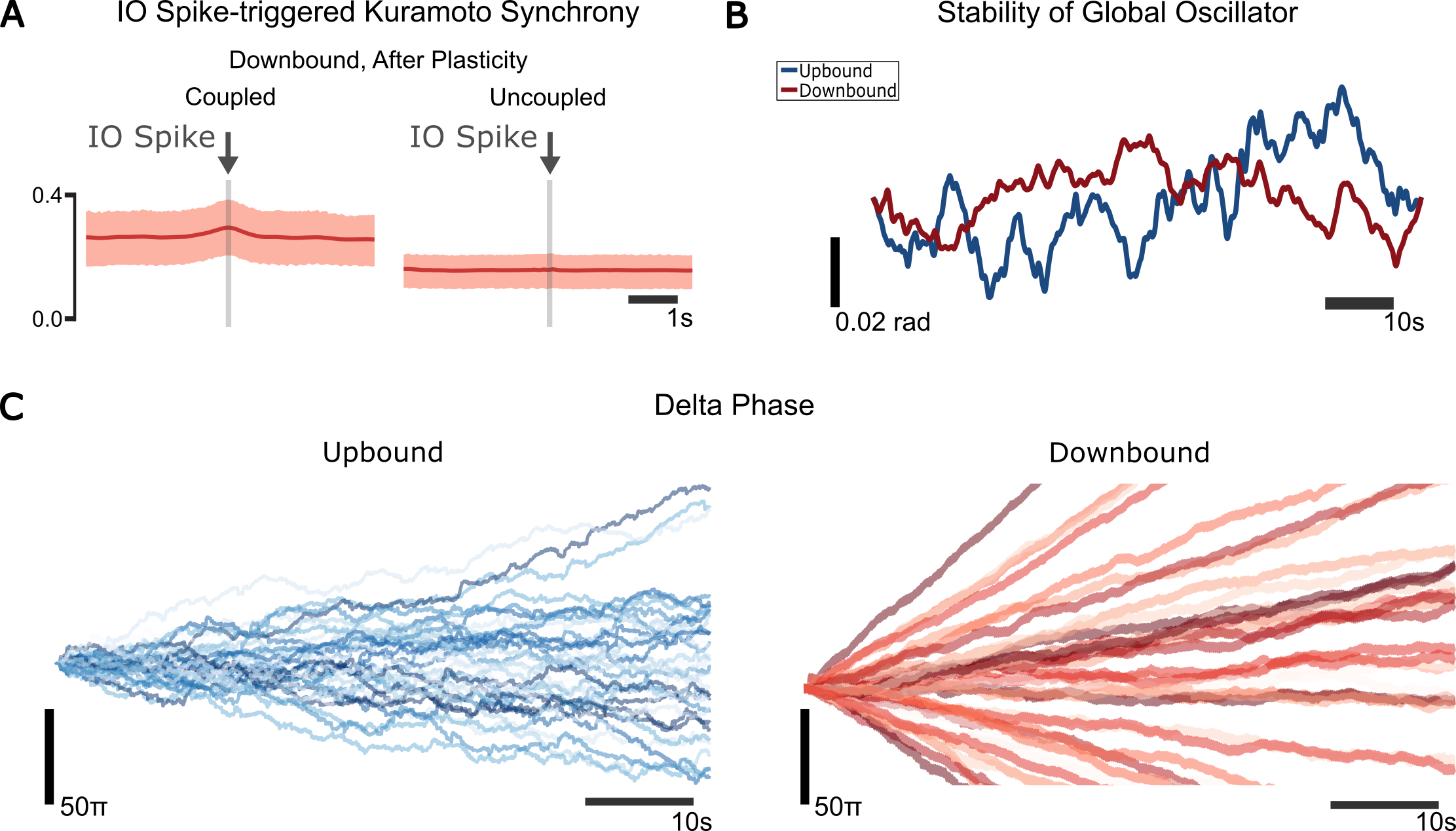

Supplement: S3 Fig — When uncoupled, the global oscillator still exhibits stable clock behavior. In Upbound zones there is less phase dispersion, but no phase locking appears in uncoupled scenarios. Downbound zone oscillators act completely independently. (A) IO Spike triggered Kuramoto synchrony with average 25/75 percentiles in Downbound, after plasticity. Average STO synchrony is higher for the coupled case. STO synchrony peaks before and after IO spikes in the coupled case, but not for the uncoupled case. This indicates that gap junctions not only set a base level of synchrony, but they also induce synchronous spikes. Note the increase of synchrony before the IO spike in the coupled case. (B) Stability of the global oscillator (see S2.1B), for the uncoupled case. Mean oscillator phase has smaller variation. (C) Delta phase plot (see S2.1C) for the uncoupled case (without modulo because phase locking does not occur as in the coupled case (S2 Fig). IO neurons in the Upbound zone still keep some synchrony while for Downbound zones, oscillators behave independently. (TIF) [file pcbi.1013609.s003.tif]

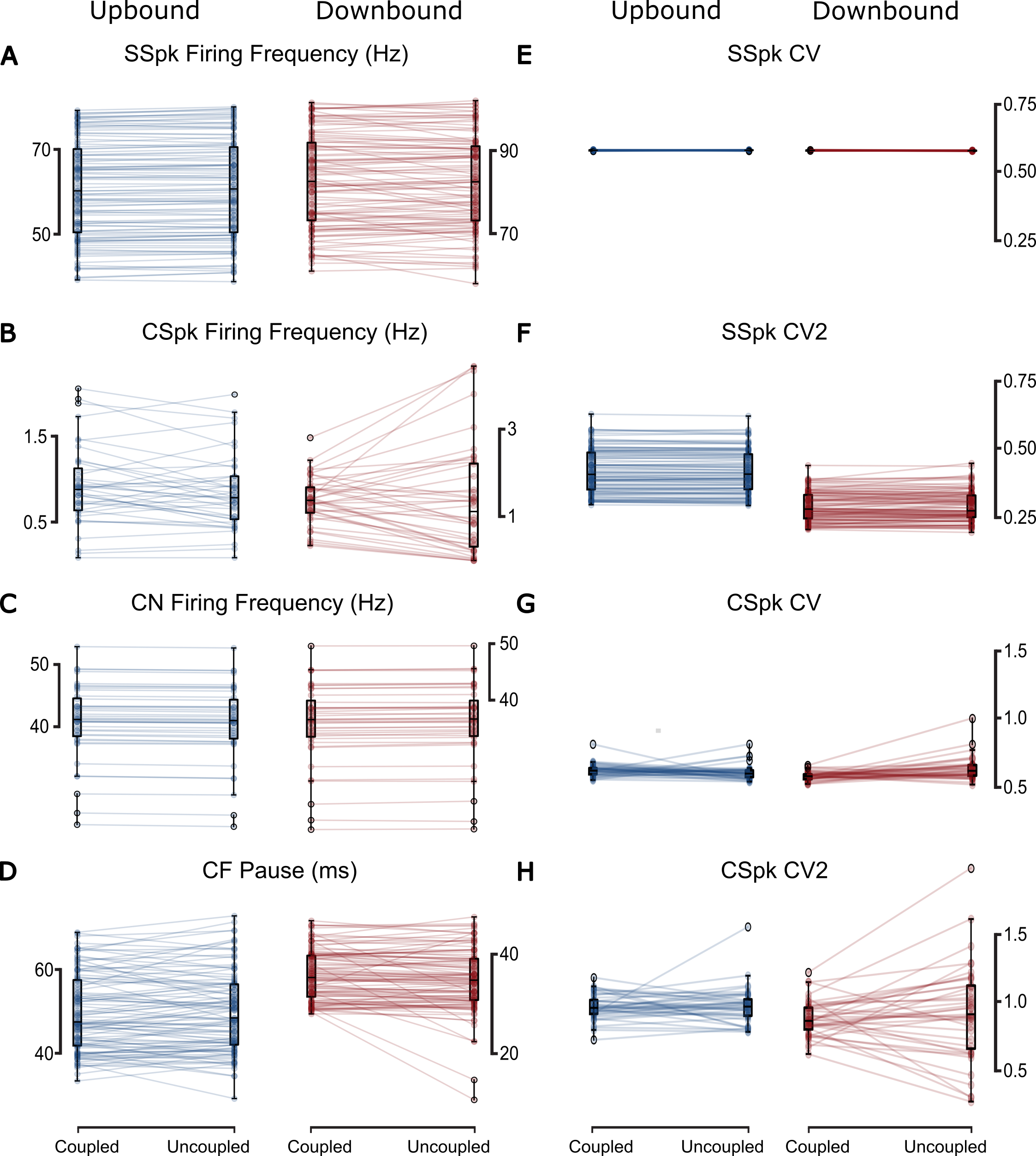

Supplement: S4 Fig — (A) SSpks frequency is significantly different across zones (coupled: t = 13.9, p < 0.001, uncoupled: t = 13.61, p < 0.001; two-sample Student’s t-test; n = 100). (B) CSpks frequency (coupled: t = 3.98, p < 0.001, uncoupled: t = 2.5, p = 0.014; two-sample Student’s t-test; n = 40). The variance of CSpks is significantly different in the uncoupled case across zones(F = 8.65, p < 0.001; F-test using CCDF of F-distribution; n = 40). (C) The CN firing frequency (coupled: t = -3.26, p = 0.002, uncoupled: t = -2.98, p = 0.004; two-sample Student’s t-test; n = 40). (D) The length of the CF Pause is significantly different across zones for both coupling cases (coupled: t = -12.52, p < 0.001, uncoupled: t = -14.39, p = 0.014; two-sample Student’s t-test; n = 40). (E) SSpk CV is significantly different for the Downbound zone across coupling cases (t = 5.94, p < 0.001; two-sample Student’s t-test; n = 100). For the coupled case, across both zones, the SSpk CV is significantly different (t = -4.27, p < 0.001; two-sample Student’s t-test; n = 100). (F) SSpk CV2 is significantly different for both coupled and uncoupled cas, across both zones, (Coupled: t = -13.06, p < 0.001, uncoupled: t = -12.69, p < 0.001; two-sample Student’s t-test; n = 100). (G) CSpk CV is significantly different for the Downbound zone across coupling cases (t = -3.69, p < 0.001; two-sample Student’s t-test; n = 100. For the coupled case, across both zones, the CSpk CV is significantly different (t = 7.16, p < 0.001; two-sample Student’s t-test; n = 100). (H) CSpk CV2 is significantly different for the coupled case across both zones, (t = -3.57, p < 0.001; two-sample Student’s t-test; n = 100). For the uncoupled case, the variance is significantly different across both zones (F = 6.86, p < 0.001; F-test using CCDF of F-distribution; n = 100). (TIF) [file pcbi.1013609.s004.tif]

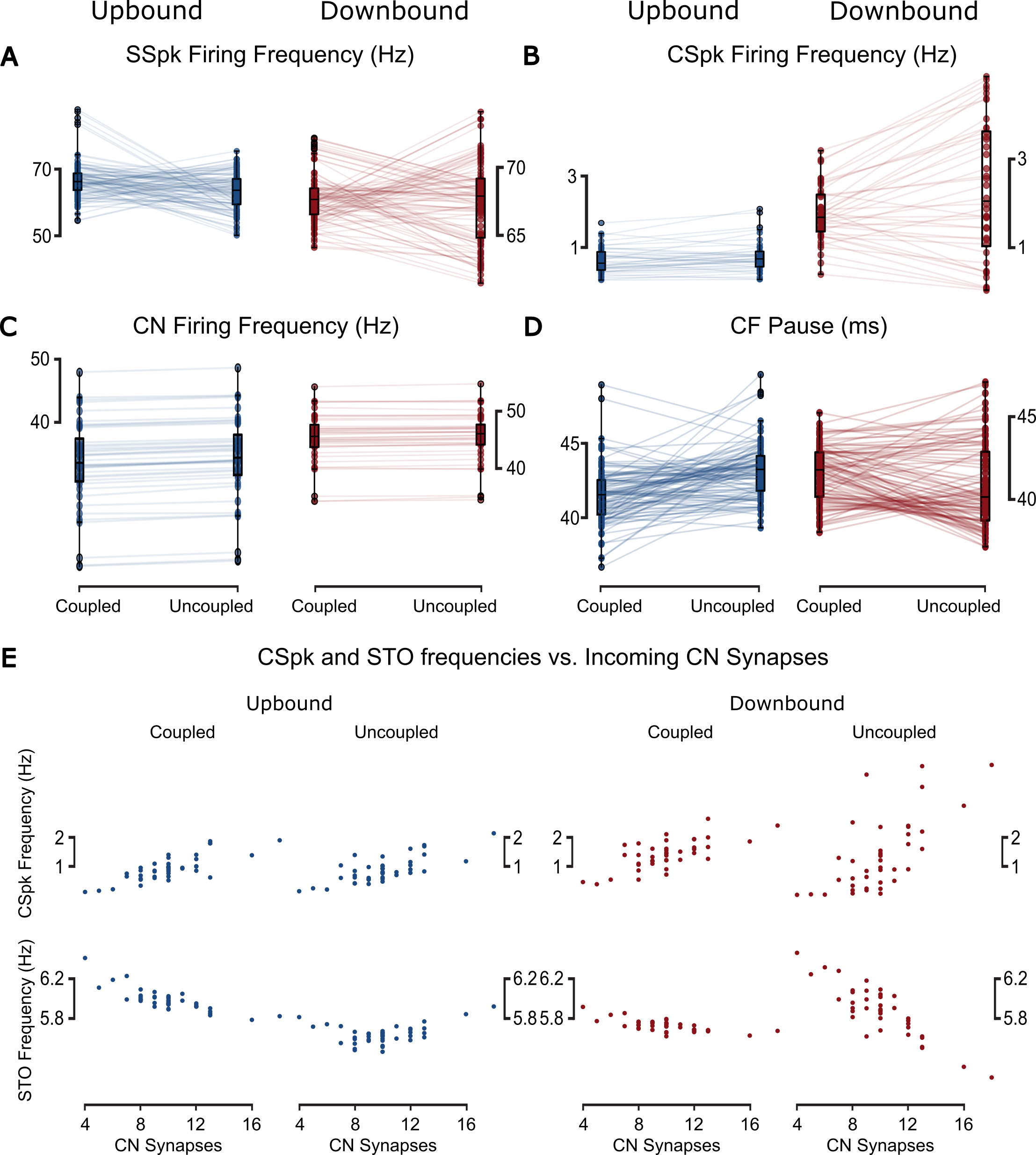

Supplement: S5 Fig — (A) After Plasticity difference for SSpks is significant across coupled cases for the Upbound zone (t = 4.51, p < 0.001; two-sample Student’s t-test; n = 100) and for coupled case across zones (t = -5.36, p < 0.001; two-sample Student’s t-test; n = 100). The variance of SSpk frequency is significantly different for the coupled case across both zones (F = 4.85, p < 0.001; F-test using CCDF of F-distribution; n = 100). (B) CSpk frequency is significantly different across coupling cases (coupled: t = 9.44, p < 0.001, uncoupled: t = 5.84, p < 0.001; two-sample Student’s t-test; n = 40) and the variance is also significantly different (coupled: F = 2.91, p < 0.001, uncoupled: F = 12.19, p < 0.001; F-test using CCDF of F-distribution; n = 40). (C) CN firing frequency is significantly different across coupling cases (coupled: t = 9.15, p < 0.001, uncoupled: t = 8.87, p < 0.001; two-sample Student’s t-test; n = 40). (D) The length of the CF pause is significantly different across coupling cases for Upbound and Downbound zones (Upbound: t = -6.25, p < 0.001, Downbound: t = 2.5, p = 0.013; two-sample Student’s t-test; n = 40). It is also significantly different across zones for the uncoupled case (t = -7.76, p < 0.001; two-sample Student’s t-test; n = 40). (E) Increase in CN-to-IO inhibitory synapses, and thus inhibition strength, led to a decrease in IO STO frequency and a concurrent increase in CSpk frequency. (TIF) [file pcbi.1013609.s005.tif]

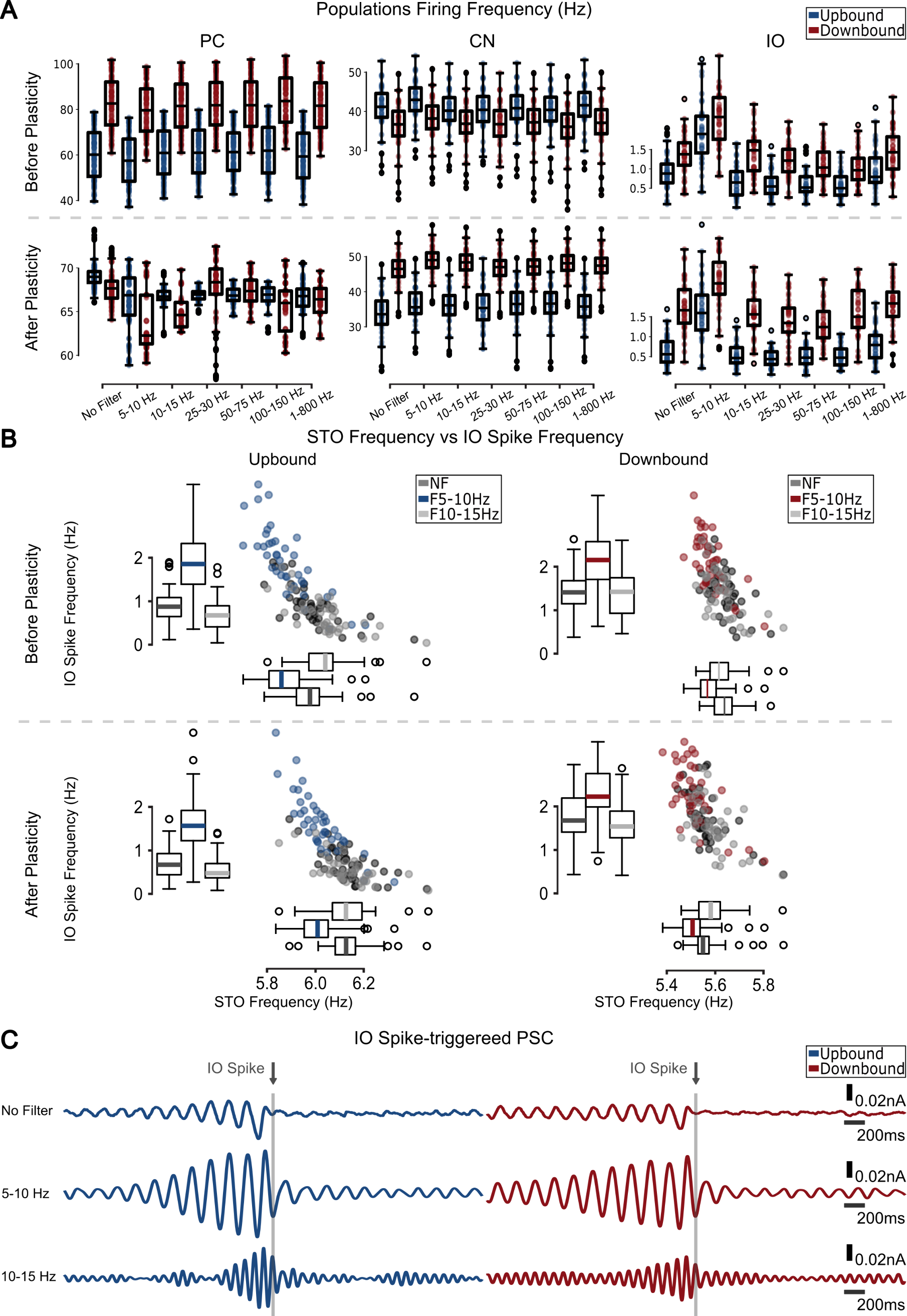

Supplement: S6 Fig — (A) Firing frequency distributions for each population before and after plasticity for different frequency bands. (B) STO frequency vs IO spike frequency for Upbound (left) and Downbound (right) zones for before (top) and after (bottom) plasticity. There seems to be an inverse relationship between STO frequency and IO spike frequency. To reduce the effect of the spike refractory period on STO frequency, STO frequency is here defined as the mean of the 25–75 percentile of the inverse of time differences between zero crossings in the Hilbert phase. For the 5–10 Hz filter in the Upbound zone, STO frequency seems to decrease while STO spikes increase. For the Downbound zone, STO frequency is lowered after plasticity, correlated to a higher baseline CSpk frequency and thus a less pronounced difference to the 5–10 Hz filter. (C) IO spike triggered PSC for NF (top), 5–10 (middle) and 10–15 Hz (bottom) input filters. In general, IO spikes for The NF case seem to derive from entrainment followed by a final larger decrease in PSC. For 5–10 Hz filter, IO spikes seem to derive purely from entrainment to the oscillatory signal, with also a much clearer (larger amplitude) template before the spike. (TIF) [file pcbi.1013609.s006.tif]

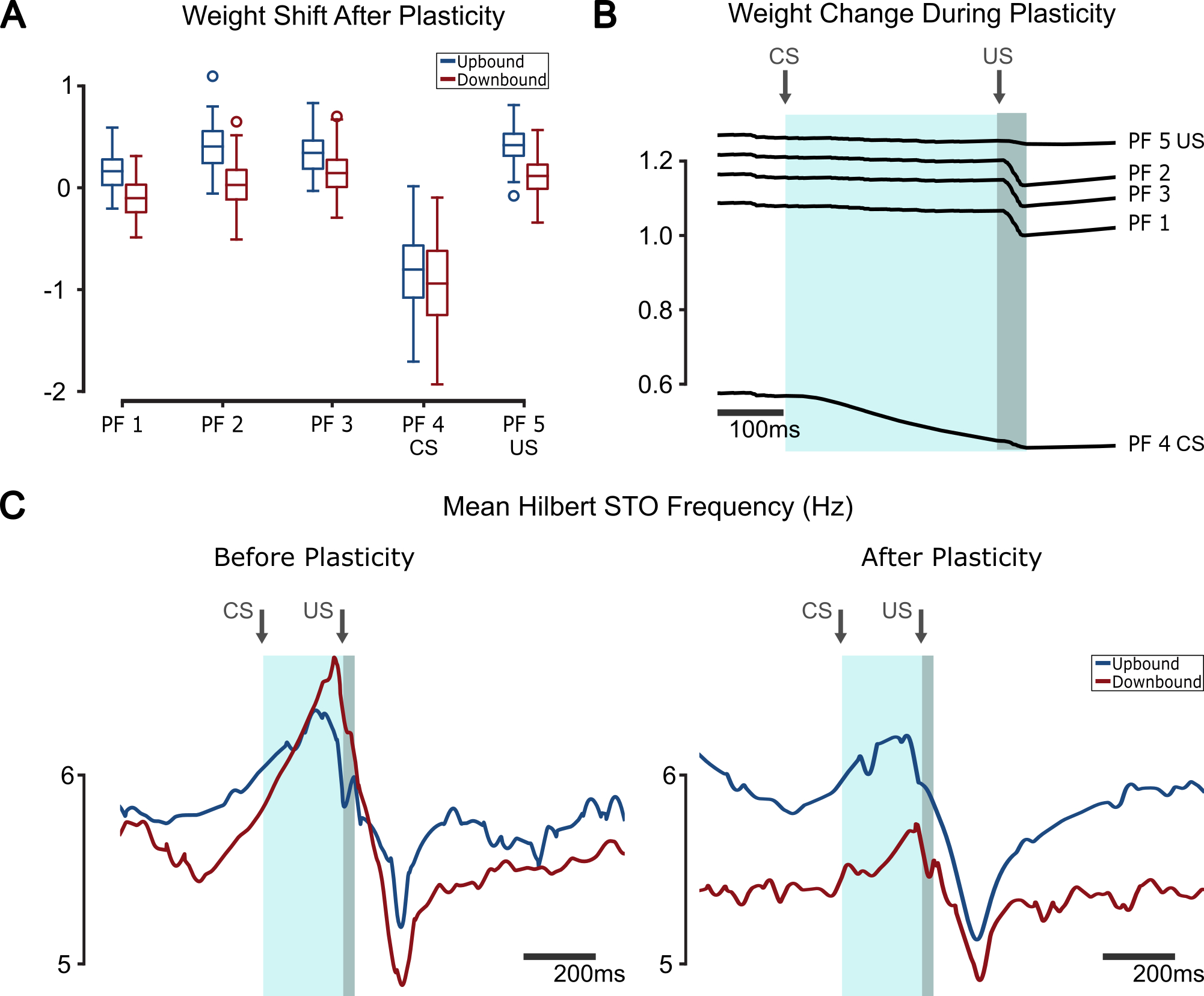

Supplement: S7 Fig — (A) Average weight shift after plasticity for each parallel fiber. The CS pf sees a large decrease in weight. (B) Weight changes during plasticity, averages for each CS/US trial. PF4 (CS) weight decreases continuously during CS. PFs 1,2,3 (noise) decrease during US and return quickly to baseline. PF5(US) responds the least to US. (C) Mean instantaneous Hilbert frequency of STOs, smoothed using 51ms 3rd order Savitzky–Golay filter. (TIF) [file pcbi.1013609.s007.tif]

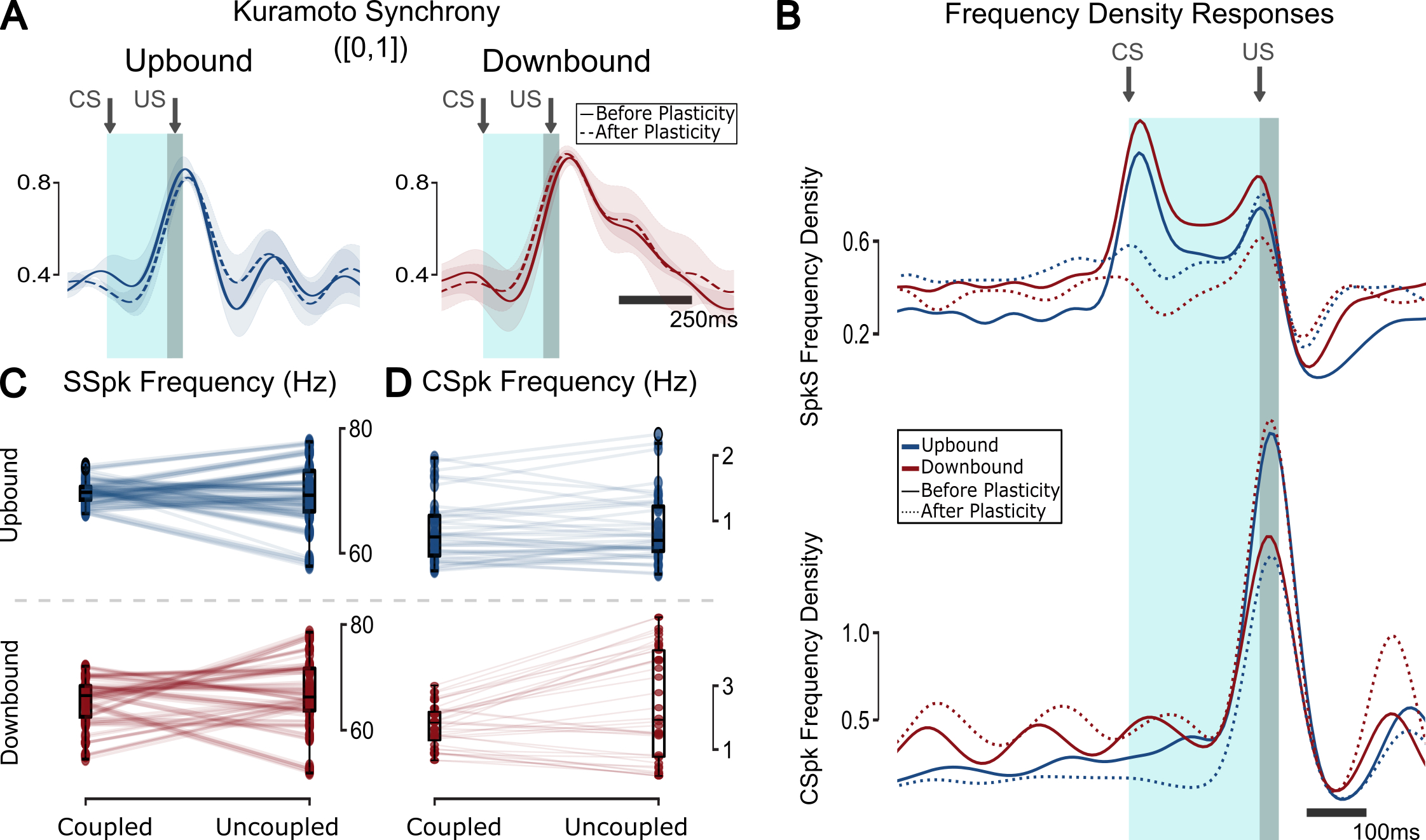

Supplement: S8 Fig — (A) Kuramoto synchrony for the uncoupled scenario (B) CS-triggered frequency density responses of the populations for SSpk (top) and CSpk (bottom). (C) SSpk frequency response after plasticity is significantly different across zones for both coupling cases (coupled: t = -8.72, p < 0.001, uncoupled: t = -3.02, p = 0.003; two-sample Student’s t-test), as well as the variance (coupled: F = 5.87, p < 0.001, uncoupled: F = 1.94, p < 0.001; F-test using CCDF of F-distribution). (D) CSpk frequency after plasticity is significantly different across coupling cases in the Downbound zone (t = -2.28, p = 0.026; two-sample Student’s t-test). It is also significantly different across zones for both coupling cases (coupled: t = 7.39, p < 0.001, uncoupled: t = -5.51, p < 0.001; two-sample Student’s t-test), as well as the variance (coupled: F = 1.83, p < 0.031, uncoupled: F = 10.4, p < 0.001; F-test using CCDF of F-distribution). (TIF) [file pcbi.1013609.s008.tif]
