# Supplementary material for: Homeostatic bidirectional plasticity in upbound and downbound micromodules in a model of the olivocerebellar loop
Source: PLoS Comput Biol. 2025 Oct 21;21(10):e1013609. doi: 10.1371/journal.pcbi.1013609 (PMC12571319; doi:10.1371/journal.pcbi.1013609)
Supplement: S1 Table — (DOCX) [file pcbi.1013609.s009.docx]

|  | Difference compared to NF  Upbound | | Difference compared to NF  Downbound | | Across Upbound and Downbound |
| --- | --- | --- | --- | --- | --- |
|  | two-sample Student's t-test | two-sample ks-test | two-sample Student's t-test | two-sample ks-test | two-sample ks-test |
| NF | ***-*** | ***-*** | ***-*** | ***-*** | ***D=0.48, p<0.001*** |
| 5-10 | ***t=8.18, p<0.001*** | ***D=0.62, p<0.001*** | ***t=10.29, p<0.001*** | ***D=0.72, p<0.001*** | ***D=0.54, p<0.001*** |
| 10-15 | ***t=11.98, p<0.001*** | ***D=0.74, p<0.001*** | ***t=11.81, p<0.001*** | ***D=0.68, p<0.001*** | ***D=0.70, p<0.001*** |
| 25-30 | ***t=13.7, p<0.001*** | ***D=0.77, p<0.001*** | *p>0.05* | *p>0.05* | ***D=0.55, p<0.001*** |
| 50-75 | ***t=12.56, p<0.001*** | ***D=0.70, p<0.001*** | *p>0.05* | *p>0.05* | ***D=0.3, p<0.001*** |
| 100-150 | ***t=11.81, p<0.001*** | ***D=0.74, p<0.001*** | ***t=6.5, p<0.001*** | ***D=0.46, p<0.001*** | ***D=0.38, p<0.001*** |
| 1-800 | ***t=10.2, p<0.001*** | ***D=0.67, p<0.001*** | ***t=5.66, p<0.001*** | ***D=0.32, p<0.001*** | ***D=0.21, p=0.024*** |
