# Supplementary material for: Homeostatic bidirectional plasticity in upbound and downbound micromodules in a model of the olivocerebellar loop
Source: PLoS Comput Biol. 2025 Oct 21;21(10):e1013609. doi: 10.1371/journal.pcbi.1013609 (PMC12571319; doi:10.1371/journal.pcbi.1013609)
Supplement: S2 Table — Upbound and Downbound modules (DOCX) [file pcbi.1013609.s010.docx]

|  | CN ***(n=40)*** | IO ***(n=40)*** |
| --- | --- | --- |
|  | two-sample ks-test | two-sample ks-test |
| NF | ***D=0.78, p<0.001*** | ***D=0.78, p<0.001*** |
| 5-10 | ***D=0.80, p<0.001*** | ***D=0.53, p<0.001*** |
| 10-15 | ***D=0.78, p<0.001*** | ***D=0.78, p<0.001*** |
| 25-30 | ***D=0.72, p<0.001*** | ***D=0.82, p<0.001*** |
| 50-75 | ***D=0.72, p<0.001*** | ***D=0.80, p<0.001*** |
| 100-150 | ***D=0.75, p<0.001*** | ***D=0.82, p<0.001*** |
| 1-800 | ***D=0.75, p<0.001*** | ***D=0.70, p<0.001*** |
