# Supplementary material for: Homeostatic bidirectional plasticity in upbound and downbound micromodules in a model of the olivocerebellar loop
Source: PLoS Comput Biol. 2025 Oct 21;21(10):e1013609. doi: 10.1371/journal.pcbi.1013609 (PMC12571319; doi:10.1371/journal.pcbi.1013609)
Supplement: Attachment — Submitted filename: Response to Reviewers.docx [file pcbi.1013609.s012.docx]

**Journal Requirements:**

1) State what role the funders took in the study. If the funders had no role in your study, please state: "The funders had no role in study design, data collection and analysis, decision to publish, or preparation of the manuscript.".

4) Please amend your detailed Financial Disclosure statement. This is published with the article. It must therefore be completed in full sentences and contain the exact wording you wish to be published.

**Reviewer's Responses to Questions**

**Reviewer #1**: In their study “Homeostatic bidirectional plasticity in Upbound and Downbound micromodules of the olivocerebellar system”, the authors develop a model of the olivocerebellar circuit with plasticity at the parallel fibre (PF)-Purkinje cell (PC) synapse, and analyze their model in two different parameter regimes of baseline activity corresponding to two distinct micromodule types. The authors show that in a regime with high PC and high inferior olive (IO) firing rates as postulated for downbound micromodules, PC and IO firing rates increase, respectively decrease over time with plasticity turned on, while they observe the opposite tendency in a regime with low PC and low (IO) firing rates as postulated for upbound micromodules.

**Reviewer #2:** This paper delivers a computational model to explore how synaptic plasticity dynamics is stabilized during cerebellar learning. The model focuses on two types of cerebellar micromodules: Upbound and Downbound. These modules differ in firing rates and plasticity tendencies: Upbound Purkinje cells (PC) tend to potentiate synaptic strength, while Downbound PCs tend to depress synaptic strength. The model thus shows that synaptic weights stabilize over time, and that IO oscillations and coupling influence the frequency ranges at which parallel-fiber inputs can be optimally stabilized. The model suggests that inferior olive driven temporal dynamics help coordinate plasticity across cerebellar modules.

**Have the authors made all data and (if applicable) computational code underlying the findings in their manuscript fully available?**

The [PLOS Data policy](https://eur01.safelinks.protection.outlook.com/?url=https%3A%2F%2Ftrack.editorialmanager.com%2FCL0%2Fhttps%3A%252F%252Fjournals.plos.org%252Fploscompbiol%252Fs%252Fmaterials-and-software-sharing%2F1%2F010f01965d9256b4-b8a71547-290f-4309-8c2e-17994f42f6c5-000000%2F8QJ9YGCVYkgcab_7t032zTmc8zU6SylFvzC14MIYV9k%3D207&data=05%7C02%7Ce.fernandezsantoro%40erasmusmc.nl%7C417672562a694efb441d08dd819d1d00%7C526638ba6af34b0fa532a1a511f4ac80%7C0%7C0%7C638809234000394313%7CUnknown%7CTWFpbGZsb3d8eyJFbXB0eU1hcGkiOnRydWUsIlYiOiIwLjAuMDAwMCIsIlAiOiJXaW4zMiIsIkFOIjoiTWFpbCIsIldUIjoyfQ%3D%3D%7C0%7C%7C%7C&sdata=RJXRhQDNKUqdFvJxzXyO2%2F8kisKqykaGfl6eflv21vU%3D&reserved=0) requires authors to make all data and code underlying the findings described in their manuscript fully available without restriction, with rare exception (please refer to the Data Availability Statement in the manuscript PDF file). The data and code should be provided as part of the manuscript or its supporting information, or deposited to a public repository. For example, in addition to summary statistics, the data points behind means, medians and variance measures should be available. If there are restrictions on publicly sharing data or code —e.g. participant privacy or use of data from a third party—those must be specified.

**Reviewer #1:** None

**Reviewer #2:** Yes

***We thank the reviewers for the careful attention they gave to our manuscript and constructive feedback. The reviewers’ suggested revisions led to substantial improvement of our manuscript and we feel that they have significantly strengthened our messages. We have edited the text throughout to address reviewers’ comments.***

***Our point­-by-­point response to comments is given below.***

**Major points**

**Reviewer #1:** While the idea of this study is interesting, I have a few difficulties with the underlying modeling logic and the presentation of the results.

- The authors motivate their study with the very general problem of stability of synaptic weights in the face of ongoing activity and continuous plasticity in recurrent networks. While it is relatively easy to understand that Hebbian plasticity in recurrent excitatory networks leads indeed to runaway activity, it is much less clear that a similar instability should exist for the olivocerebellar loop, in particular as no recurrent excitation (even if via intermediate populations) is involved.

*We apologise for not being clear about our modeling choices and questions in the introduction. We have substantially revised the presentation of our reasoning (lines 110-118).* *In the introduction we motivated the study against the backdrop of Hebbian plasticity runoff of weights, but indeed, our question relating to weight stability is more subtle as it relates to specific weight distributions induced by activity dependent plasticity over longer durations. As the reviewer correctly points out, we "bake in" stability via the BCM rule. This is because the driving questions are about the changes of a system that is, to a large extent, slave to its own output. The main question in our study is “What are the balancing changes in the activity of the population and input weights provided stationary inputs?”. We have adapted the introduction to sharpen the presentation of the actual question being handled.*

- To address the issue of weight homeostasis, the authors study the effect of repeated presentations of relatively long (120s) bouts of frozen random input while synaptic plasticity operates, and probe activity and weights with synaptic plasticity being switched off. It is not clear how this numerical experiment, which somewhat resembles more ‘classical’ learning scenarios with repeated inputs, relates to the problem of ‘ongoing’ plasticity in the absence of any specific learning. Do results depend on the duration of the frozen input? Do results change when the input is not frozen? Is the activity different while plasticity is ‘switched on’?

*We thank the reviewer for these questions, which we now address in the results (lines 161-170) and discussion (lines 625-633). In our study, the “frozen noise” refers to the use of a fixed realization of a stochastic Ornstein-Uhlenbeck (OU) process, generated once and reused across simulation epochs. This input retains temporal variability — it is not a repeated or static signal within a trial — but its use across conditions allows direct comparisons under matched input statistics. The purpose was not to simulate classical learning with repeated identical stimuli, but rather to examine how the circuit responds to stationary, yet complex and time varying input. Our choice was motivated by in vivo experimental data of granule cell and PF activity during learning, at rest and during walking (Giovannucci, Badura et al. 2017; Lanore et al 2021; Lee et al. 2023), which shows complex, high dimensional firing patterns transmitted into PCs in awake animals.*

*Our goal was to test whether synaptic weight distributions would stabilize under such temporally structured but statistically stationary input. We observed that weights converged to a stable distribution after an initial transient and remained bounded across repeated epochs. Using different realizations of OU noise yielded similar convergence, with little variability due to input difference. Importantly, weight stabilization did not depend on the input duration, it only influenced the speed at which synaptic weights settled into a new equilibrium, without altering the steady-state outcome.*

*Finally, we used plasticity-on epochs to allow weights to evolve under this fluctuating input and then probed the network dynamics with plasticity turned off. This ensured that the activity during the probe reflected the stable state of the circuit without additional synaptic modifications. When plasticity was active, the BCM and CSpk-triggered LTD mechanisms allowed weights to gradually adjust to a new homeostatic equilibrium. Hence, we focused our comparisons on before and after the plasticity epochs, as activity during plasticity would otherwise reflect an evolving, non-stationary regime in which the weights change continuously. After an initial epoch transient, the weight changes in subsequent epochs became minimal — further updates were small relative to the absolute weight values. We have clarified this point in the revised text.*

- The homeostasis is very much ‘baked in’ in the BCM plasticity rule, as the authors themselves concede. It is therefore not very surprising that synaptic weights stabilize (in addition to the first comment). The proposed BCM rule is furthermore very generic, and not constrained by experimental data on synaptic plasticity (which admittedly is limited despite a relatively huge literature on plasticity rules measured in slice, see my comment below on the cited literature). This modeling choice therefore contrasts with the declared aim of studying a biologically detailed model of the olivocerebellar system. A question would be whether the observed effects (some of which being rather small) depend on parameters of the plasticity rule?

*We appreciate the reviewer’s critical perspective on the use of the BCM rule. We would like to clarify that the plasticity rule was not designed to generate our findings; rather it was selected because it satisfies three biologically motivated constraints that we deemed essential for modeling cerebellar PF-PC plasticity under ongoing input:*

*(1) it exhibits a plateau that prevents runaway potentiation or depression;*

*(2) it operates at physiologically plausible PC firing rates where the only constraint is that potentiation cannot occur above 250 Hz (pathological firing rates); and*

*(3) it is sensitive to relative timing between PF and CSpk activity.*

*Among available formulations, a BCM-like rule is the simplest and most interpretable rule that meets these requirements. Within this framework, homeostasis is an expected feature, not an artifact; it allows the model to maintain stable activity in the presence of reverberating loop dynamics, which was one of the central aspects we wanted to investigate.*

*As the reviewer notes, some degrees of freedom remain in this formulation, particularly the choice of the*

*threshold time constant. While the rule itself constrains the qualitative shape of the plasticity curve, the time constant is not directly specified by experiment. Nonetheless, we based our selection on the typical duration of CF pauses (10-20 ms). To address the reviewer’s concern, we now explicitly report in the revised Methods (lines 919-928) that we tested several values of threshold time constant across this plausible range. We found that the overall behavior of the model remained robust across parameter variations. However, there was a narrow range of threshold time constant values for which the direction of plasticity of both modules aligned with experimental observations: net depression in Downbound modules and net potentiation in Upbound modules.*

- Getting back to the issue of ongoing plasticity and homeostasis vs. a specific learning scenario: In this work, the so-called downbound modules turn out to have lower baseline firing rates than the so-called upbound modules once plasticity has been allowed to operate. Conceptually, I would have expected these modules to be distinguishable with their respective characteristics after plasticity, or rather while plasticity operates, at least if homeostasis in the absence of any specific learning is addressed. Especially in light of this, the authors’ conclusion that the feedback loop establishes preferred activity levels for microzones (ll. 505-508) seems a bit far-fetched, as simple spike rates are actually inverted.

*Our use of Upbound and Downbound terminology reflects the initial intrinsic firing properties of PCs in each module, based on zebrin identity, rather than a fixed post-plasticity outcome. This terminology is based on (Zhou et al. 2014; De Zeeuw 2021; Voerman et al. 2022). When plasticity is activated, the feedback loop reconfigures firing rates as a consequence of both BCM and CF-triggered plasticity. While this reverses the baseline firing asymmetry (with a very small difference), it does not contradict the concept of preferred activity levels. Instead, it highlights that these levels emerge through the closed-loop dynamics, where homeostatic input-driven forces interact. We have clarified this distinction in the manuscript (lines 101-107).*

- The authors deliberately choose to focus solely on inhibitory signaling from the CN to the IO, despite acknowledging in the discussion that the excitatory pathway may be important to consider. MF input further directly arrives at the cerebellar nuclei, which might also shape the dynamics of the micromodule feedback loop. It is unclear whether the results of the present study would hold when more elements of the circuit are taken into consideration.

*We thank the reviewer for raising this important point. Our decision to focus on the inhibitory CN-to-IO projection was motivated by the need to isolate and study the fundamental reverberatory dynamics of the olivocerebellar loop. While this simplification excludes other biologically relevant pathways, such as the excitatory CN-MDJ-IO pathway and MF collaterals to the CN, our model still captures core mechanisms underlying plasticity stabilization through IO resonance and feedback, which has not previously been modelled.*

*We fully agree that these additional pathways (particularly the excitatory CN-IO projections via the MDJ) are important modulators of IO excitability and their inclusion in future models could help refine hypotheses about functional modular segregation. Regarding MF collaterals to the CN: these are known to provide excitatory input that could modulate CN firing rates independently of PC inhibition. However, we expect that PC-driven inhibition remains the dominant mechanism shaping IO timing in the context of the CF-triggered pause. While MF input could influence baseline CN excitability and temporal filtering, we do not anticipate it would override the core effect of the PC-CN-IO inhibitory loop, particularly in the context of timing precision and phase-resetting of IO STOs.*

*Our model focuses on a minimal closed-loop motif which we see as a baseline scenario. We anticipate that adding these pathways would allow for modulatory influences and richer plasticity dynamics, but not invalidate the core findings. We now explicitly acknowledge these pathways and their likely roles in the Discussion (lines 664-676) and highlight them as important directions for future expansion of the model.*

**Reviewer #2:** The paper provides a tool to test different hypotheses of interest for those researchers working on cerebellar function and learning. I find several results of this paper relevant for publication. However I also find several weaknesses:

1-This is a rather lengthy paper hard to read because of its organization. A minimal explanation of the model is required to understand its assumptions, the provided synaptic plasticity exploration and the results. However key model descriptions come at the very end of the paper, and thus readers unfamiliar with the authors' work will be completely lost. The most notorious example of this is the use of the term "synaptic weight" in this model, which is first explained on page 40, but it is used from the very beginning.

*We apologize for such oversights, and we are grateful to the reviewer for the attentive eye. We agree that the organization of the manuscript could be improved for accessibility, particularly for readers unfamiliar with our earlier work. In response, we have now added a concise description of the model architecture and synaptic plasticity assumptions earlier in the manuscript, immediately following the introduction (lines 126-145). This includes a brief explanation of what is meant by “synaptic weight” in our framework, to ensure that readers can interpret this term correctly. The full mathematical implementation remains in the Methods for completeness, but key concepts are now introduced earlier to improve clarity.*

2-The paper uses very simple adaptive exponential integrate and fire models to describe PCs and cerebellar nuclei (CN) and a three-compartment biophysical description of the IO cells. An explanation is required why PC morphology is not an issue for the conclusions reached in this model, as well as the simple dynamics for all synapses beyond the plasticity models used.

*The modeling choices reflect the specific focus of our study on the role of IO-coupled dynamics in shaping plasticity and stability in the olivocerebellar loop. For this reason, we chose to model the IO neurons in greater biophysical detail, including dendritic compartments and gap junction coupling, to capture the subthreshold oscillations important for olivary synchrony and climbing fiber timing.*

*In contrast, the PC model was intentionally kept minimal — using an adaptive exponential integrate-and-fire neuron — because the important feature needed to test our hypothesis is its firing output in response to synaptic input, which is captured in the quasi linear relationship in the IxF response curve. Detailed morphology becomes important if we were aiming to study non-linear dendritic responses and ionic dynamics would become relevant if we were considering mechanisms of intrinsic plasticity, for which there is scarce experimental data. The goal was to capture how PC output is shaped by PF input and IO-mediated pauses, which are sufficient for triggering plasticity through a BCM rule that depends on SSpk rate (PF input) and CF timing. We do not model calcium spikes or dendritic integration explicitly, as these are not required to test our hypothesis about loop-level resonance and synaptic weight stability. Similarly, the CN neurons are modeled using the same type of model to capture their rate response for the inhibitory feedback to the IO.*

*While more complex PC models could, in principle, refine microcircuit dynamics, our simple model strikes a balance between biological plausibility and interpretability. Nonetheless, we acknowledge that future work should incorporate simplified multicompartmental PC models (e.g. with 2-3 dendritic compartments) to more explicitly address calcium dynamics and dendritic integration in different branches without adding prohibitive complexity.*

*We have added this rationale for model complexity in the Methods in a new subsection (lines 743-758).*

3-Stochastic input should be better justified for the purpose of the plasticity exploration, particularly in a system where functional input timing and correlations can be essential for the learning process.
*As explained in the response to Reviewer 1, the use of the stochastic input in our model was a deliberate design choice aimed at investigating precisely input timing and correlations, without having to assume a particular functional source. As the input across different epochs is repeated, the network is bound to be exposed to particular statistical regularities, as they come from a seeded stochastic source. The question posed is whether plasticity leads weight distributions to converge under any stationary, as long as there are specific temporal relationships in the inputs. In particular, we wanted to explore how the dynamics of the olivocerebellar loop, rather than the specific temporal structure of the external input, shapes learning outcomes.*

*The OU process used for PF inputs simulates temporally correlated but unstructured synaptic drive, intended to reflect the integration of many asynchronous excitatory inputs during ongoing sensorimotor activity. While the OU process contains a broad range of frequencies, its autocorrelation properties make it more biologically realistic than uncorrelated white noise, while still remaining analytically and computationally tractable. We add this explanation to our manuscript (lines 161-174)*

4-In general, it is not clear what results required fine tuning in the model, and what results are robust for a wide enough parameter space. Hypotheses are drawn but then the model seems to be tuned to lead to the phenomena hypothesized.

*We thank the reviewer for this apparent key issue. To clarify, we did not fine-tune the model to produce the hypothesized phenomena (e.g. synaptic weight stabilization or frequency-specific IO resonance). Instead, we tuned baseline model parameters to match experimentally observed firing statistics under spontaneous activity conditions —in particular, PC SSpk and CSpk firing rates, CN activity, and IO rates and oscillation frequencies, based on the experimental data (Fig. 3). Once the model was constrained to operate within the physiological regimes, all results related to plasticity and loop dynamics (weight convergence, CF pause, frequency band selectivity) emerged from the interaction of BCM plasticity, IO coupled, and intrinsic neuronal dynamics, without further adjustment. We now clarify this distinction in the Results (lines 180-186).*

5-Overall, limitations of the modeling approach beyond what the model left out should be discussed in the view of the conclusions.

*We thank the reviewer for pointing out the need to more critically assess the limitations of our modeling approach in light of the conclusions drawn. While our model deliberately excluded several known features of the olivocerebellar system — such as excitatory MDJ input to the IO, cortical and subcortical feedback, and the full diversity of PC microzones — this was intended to isolate the contributions of the core loop architecture and intrinsic cellular dynamics. We agree, however, that the use of a generic BCM plasticity rule, while effective in capturing key homeostatic interactions, lacks direct experimental constraints from experimental plasticity data. Similarly, the stochastic PF input used in our simulations does not capture all features of mossy fiber activity during natural behavior, including possible bursts or structured temporal sequences. Furthermore, we have added a note on how the frequency band near 10 Hz (which we found to preferentially drive IO responses) may reflect Golgi cell-induced frequency gating, which needs further investigation. We now discuss these limitations explicitly in the revised discussion in a new subsection (lines 704-729).*

**General comments**

**Reviewer #1:**

- It would be helpful if the authors provided more context on the concept of upbound and downbound microzones, e.g. cite original work on individual zones and point explicitly to review introducing the concept that these zones represent distinct modules, potentially acting in concert (ll. 272-274 are unclear in this regard).  
*We thank the reviewer for this valuable suggestion. We have now clarified the concept of Upbound and Downbound microzones and added historical context and key references in the introduction (lines 101-107). We now also discuss zone-specific modulation of PC firing in the context of Figure 3 data.*

- Fig. 3A,B: The value of the comparison to experimental recordings during eyeblink conditioning is not clear (also the formulation in l. 279 is misleading as the paper doesn’t feature experimental recordings itself). First, as the authors note themselves, the timescales of the expressed changes in firing rate are incompatible. Second, in eyeblink conditioning, the changes appear over many trials, and are expressed relative to the timing of an unconditioned stimulus. It is not clear (and not described in the methods) how the changes arise in the model firing rates. Third, eyeblink conditioning is a specific learning paradigm (with specific error signals conveyed via the IO) and insofar much different from the questions the authors initially set out to address, namely weight homeostasis in the presence of ongoing plasticity.

*Our model does not aim to simulate the trial structure or stimulus-specific learning mechanisms of eyeblink conditioning. Our goal in comparing this experimental data was not to reproduce the behavioral learning paradigm, but rather to explore whether our homeostatic plasticity framework could give rise to directionally consistent firing rate changes in Upbound and Downbound zones —as observed during learning in cerebellar associative tasks.*

*To avoid confusion, we now clarified in the text and figure caption that Fig. 3A is a sketch based on published data rather than an experimental trace. We also explicitly acknowledged the difference in timescales, noting that plasticity in eyeblink conditioning emerges from many trials and is aligned to a conditioned stimulus, whereas our model uses frozen input to study convergence of synaptic weights under internally generated dynamics.*

*Despite these differences, we find that the same Upbound-Downbound polarity in SSpk modulation emerges from our model without fine-tuning, and this suggests that intrinsic firing properties and loop dynamics alone can impose directionality on plasticity —a result that may generalize beyond specific task structures. We have updated the figure caption and the Results text accordingly (lines 339-354).*

- The references are at times unnecessarily exhaustive if not mistaken, while other parts of the literature are rather superficially touched upon:

*We thank the reviewer for these points. We address them below.*

- The first 3 refs. seem a bit off as far as they are meant as an introduction to sensorimotor plasticity.
*We thank the reviewer for pointing this out. Indeed, the referencing in the introduction could be more clearly aligned with the text. We have now revised the opening paragraph (lines 53-58) to more clearly separate the general background on sensorimotor plasticity from the introduction of the olivocerebellar loop. The citations [1-3] now specifically support the general principles of plasticity in sensorimotor loops, while references to the cerebellar literature begin with [4]. This change also improves the clarity of what set of references is intended to support.*

- The “ceiling and saturation effects” referenced on l. 80 are unrelated to the specific limitations of PF-PC synaptic plasticity that has been hypothesized to support only LTD in the presence of CF signals. In contrast, the discussion of PF-PC LTD is pretty succinct if not vague (l. 82 “when PFs and CFs are activated in the same period”), referencing relatively old reviews. The picture might actually be a bit more complicated given the observed heterogeneity in PF-PC plasticity (e.g. reviewed in ref. 26), and even inverse to the classical picture of PF+CSspk = LTD, see Zang & Schutter, Curr Opin Neurobiol 2021; Bouvier et al., eLife 2019; Titley et al., J Physiol 2019.

*We agree that the previous description presented an overly simplified view. We have now revised the relevant paragraph in the introduction (lines 79-87) to better reflect the current understanding of PF-PC plasticity, incorporating recent findings and citing Zang Schutter 2021, Bouvier et al. 2019 and Titley et al 2019 which present compelling evidence for heterogeneous and context-dependent plasticity dynamics. We also clarified the role of homeostatic mechanisms in preventing saturation of synaptic weights.*

- l 141 is an example of where 10 refs are provided for a single statement, which include a couple of modeling papers despite the statement being about experimental findings. Ref 58 furthermore seems not to be related to the referenced statement (IO phase precessions).

*We thank the reviewer for this helpful observation. We agree that the reference list in line 141 was overly exhaustive and included citations that were not directly relevant to the specific claim about IO phase modulation. We have now limited the references in that sentence to the most relevant experimental studies. We have also removed ref 58 which was not appropriate for that context.*

- Fig. 4: While in A the effects of plasticity look relatively minor, they look indeed stronger in B. This is interesting, as there are not plastic changes between CN and IO. Could the authors try to explain this observation? A related observation is that IO firing rates increase (decrease) in downbound (upbound) zones after plasticity despite an increased (decreased) activity of their inhibitory CN inputs, which is a rather counterintuitive result that the authors do not comment much on. (There is also evidence from optogenetic stimulation experiments that increased PC activity leads to increased IO discharge, Chaumont et al., PNAS 2013.) To explain their observation, in the subsequent section the authors state that “slowing down the STO creates more opportunities for spikes”. Could the authors expand on this mechanism?

*We thank the reviewer for the perceptive comment. We agree that the plasticity effects are more prominent in the CN-triggered IO response (Fig. 4B) than in the PSC-triggered view (Fig. 4A). This reflects the fact that plasticity in our model occurs only at the PF-PC synapse, and therefore modulates CN activity indirectly via changes in PC firing. The CN, in turn, affects the IO. Thus, the plasticity-dependent changes seen in Fig. 4B emerges from the loop-level dynamics, not direct synaptic modifications between CN and IO.*

*Regarding the second observation — the apparent paradox of increased IO firing in the Downbound module despite increased CN inhibition — this behavior emerges from the interaction between IO STOs and the timing of inhibition. Stronger inhibition can slow down the STO, which paradoxically increases the window for excitability near the next oscillation. This mechanism leads to a higher probability of IO spiking, consistent with observations in our simulations and supported by the hypothesis described in Fig. S4E. In contrast, in the Upbound case, weaker inhibition leads to less slowing of the STOs and thus, reduced IO excitability. This counterintuitive mechanism is consistent with previous findings that IO resonance and phase-resetting are highly sensitive to the timing and strength of inhibitory input. We now expand the explanation of this result in the text (lines 403-409) and refer to Chaumont et al, whose results together with ours emphasize the emergent and non-linear nature of the loop dynamics and highlight how subtle shifts in network resonance can significantly affect IO output.*

**Minor points**

**Reviewer #1:**

- Sentence ll. 120-128 resuming the findings is difficult to understand; better try to break into clear statements.
*We agree that the original formulation was overly long and complex. We have revised this section to present the findings as clear, concise statements, making each point easier to follow. The new version separates the key conclusions regarding stabilization, loop dynamics, frequency selectivity, and the role of gap junctions.*

- l. 168 incomplete sentence

*We have revised the text to ensure the sentence is grammatically complete and logically integrated with the surrounding discussion of IO cell dynamics.*

- Abbreviation PSC is not explained.

*We have now defined the abbreviation PSC (postsynaptic current) upon its first mention in the main text (lines 135-136) and when discussing the results of Fig. 4 (lines 386-388). Additionally, we clarified that in our model the PSC represents the total current arriving at the PC from the PF (multiplied by their synaptic weights and summed).*

- The terminology “BCM & CSpk weights” is unclear/misleading (they describe weight changes if I understood the model correctly).

*We agree that the phrase “BCM & CSpk weights” was unclear. What we meant to describe were the two plasticity contributions to synaptic weight change: one governed by the BCM rule (bidirectional, continuous) and the other triggered by CSpks (event-driven LTD). We have revised the terminology throughout the text and figure caption to clarify that these refer to plasticity components contributing to the total change in synaptic weight, not to separate weight variables.*

- Are the hard weight constraints reached? If yes, by what fraction of weights?

*We thank the reviewer for this important question. In our model, the only hard constraint is on the PC firing rate, which is limited to 250 Hz to avoid pathological spiking. However, no hard bounds are imposed on synaptic weights themselves. Instead weight dynamics are regulated through a homeostatic BCM mechanism with a sliding threshold combined with a CSpk-triggered LTD mechanism.*

*While the weights are not explicitly bound, we tracked the evolution of the PC activity during simulations and found that they remained within a physiologically plausible range (>20 Hz and <160 Hz). We now clarify this point in the Methods (lines 914-918).*

- Fig. 2C: IO spiking seems pretty bursty - did the authors characterize this burstiness and can compare it to experimental data?

*Fig. 2C indicated the (action potential) “threshold”. Now it is explicitly written “action potential threshold”. The burstiness observed in Fig. 2C was intentionally emphasized in this trace to illustrate, within a short time window, the interaction between IO activity and both the BCM and CSpk-triggered LTD components of plasticity. This trace was selected to highlight the immediate effects on synaptic weight change for different spike intervals, rather than to reflect the full range of IO dynamics. Our neurons exhibit diverse temporal profiles, including tonic firing, burst periods, and quiescence. The average population activity (~1 Hz,* ***Fig. 1B****) is consistent with experimental observations. The IO neuron population has a distribution of firing rates as shown in Fig. 3B. We did not explicitly tune the burstiness of individual IO neurons. Instead, output arises from network and input dynamics, including CN feedback and gap junction coupling. The burst frequency ranges never exceeded 6 Hz, consistent with experimental observations, this is shown in the figure below. We have now clarified this point in the caption of Fig. 2 and added the CV and CV2 results.*

- How much do results depend on synaptic delays? They can be expected to vary, and are not known with ms precision (much shorter times have been reported for PC-CN connections, ref. 43).

*The exact timing of synaptic delays — particularly in the PC to CN pathway — can indeed vary and is not precisely known. We did not systematically vary the delays in a dedicated experiment, and thus do not claim robustness across a wide parameter sweep. However in earlier development stages of the model, we initialized the model with much shorter synaptic delays: 3-5 ms. This previous iteration of the model showed the same main qualitative findings —including the frequency preference, and PF-PC weight stabilization— were preserved, and that the phase relationships between PC, CN and IO activity remained intact. Based on this, we believe that the model is not overly sensitive to small variations in delay values, provided they stay within a biologically reasonable range (3 - 50 ms). We have now clarified this point in the Methods (lines 878-881).*

- How much do results depend on BCM rule parameters? Did the authors perform some kind of sensitivity analysis?
*In our study, the BCM rule was implemented with parameters selected to reflect biologically plausible dynamics and maintain homeostatic balance across the population. While we did not conduct an exhaustive parameter sweep, we did test the model’s robustness by perturbing key BCM parameters —such as the time constant of the threshold. These small perturbations within the biologically plausible range did not qualitatively alter the main results of the study, including the stabilization of PF-PC weights, the divergence between Upbound and Downbound modules, or the frequency-dependent nature of CSpk generation. They only affected the convergence speed toward the new homeostatic regime. We now clarify this point in the manuscript.*

- Fig. 1A is unclear / very difficult to understand.

*We thank the reviewer for this helpful comment. In response, we have revised Fig. 1A to improve clarity. The updated version now clearly distinguishes between projection (P) and convergence (C) ratios at each stage of the olivocerebellar loop. Each neuronal population is clearly labeled, and the connections are annotated to indicate both how many targets each presynaptic neuron projects to (projection) and how many sources each postsynaptic neuron receives input from (convergence). This should make the network architecture clearer for the reader. We have adapted the caption accordingly.*

- The notion of “oscillating” weights is unclear. Do these weight oscillations depend on the length of the stimulus w/ plasticity (120s)? Is there a stable weight distribution for ongoing stimulation (w/o repeats of frozen noise)? (Those oscillations look small relative to the absolute weights btw.)

*We thank the reviewer for this valuable comment. In our manuscript, “oscillating weights” refer to small, ongoing fluctuations in synaptic weights observed after the initial convergence period. These weight oscillations do not reflect large-scale instability but rather minor adaptations that persist due to the continuous interaction of BCM potentiation and CSpk-triggered LTD in response to stochastic input. While shaped by the statistical structure of the input, these dynamics do not depend on the duration of stimulation. We observed similar fluctuations whether the input lasted 30, 60, or 120 seconds. These fluctuations were small relative to the absolute weight values and did not accumulate over time. Thus, the weight dynamics reflect a steady-state regime governed by the input statistics, not by the specific length of stimulation. We have clarified this point in the revised manuscript (lines 269-274).*

- Fig. 4: title: It seems the resonances are zone-dependent also before plasticity; slightly misleading graphics as Cspk-triggered data are aligned but have different x-axis.

*We agree that zone-dependent differences in resonant dynamics are already evident prior to plasticity, and have updated the title accordingly to avoid implying these differences only emerge after learning. Additionally, we acknowledge that the x-axes in panel A could be misinterpreted as directly comparable; while all traces are allowed to the CSpk event, the CF pause time window differs slightly for clarity. We have now clarified this in the figure caption and ensured the alignment is explicitly indicated.*

- ll. 357+ suggests that the effects reported in Fig. 4 are not caused by the entire feedback loop, is this true?
*The effects reported in Fig. 4 do, in fact, depend on the dynamics of the entire feedback loop. All the changes are shaped by the interaction between PF input, PC, CN and IO. The frequency-dependent responses described in lines 357+ and shown in Figure 5 build upon this by examining how different PF input bands modulated those loop dynamics. We have now revised the text (lines 455-459) to clarify that the observations in Fig. 4 emerge from closed-loop processing and are not limited to local synaptic mechanisms.*

- ll 370/71: What does frequency-band dependent variation with frequency mean?

*Our intent was to describe how the distribution of PC firing rates changed in response to different PF input frequency bands, and how this modulation differed between Upbound and Downbound zones. We have now revised the text (lines 462-463) to clearly state that different PF input frequencies evoke distinct patterns of SSpk and CSpk activity across the population.*

- Fig. 6: “phase period” unclear. “zebrin positive/negative” → better use previous terminology of down/upbound zones. “NF” → provide “no filter” in legend for better readability.

*We thank the reviewer for pointing out that these terms are unclear. The phase period refers to the period between zero crossings in the Hilbert analytical phase, which can happen both with regular STOs or under spikes, the latter which we want to filter out. The inverse of the phase period thus refers to the frequency of the STO. For clarification, we have updated the caption of Figure 6 as follows:*

*[...], STO frequency is here defined as the mean of the 25-75 percentile of the inverse of time differences between zero crossings in the Hilbert phase. [...]*

- ll 577-578: It is surprising that variability in IO spike rates disappears in the absence of gap junction; Fig. 7 furthermore seems to suggest the contrary. Am I missing something?

*We thank the reviewer for pointing this out. Indeed, our original phrasing overstated the uniformity of the effect. We have now revised the discussion to clarify that the loss of IO spike variability upon uncoupling is most prominent in the Upbound module, where CN inhibition is stronger and IO spike entrainment is more dependent on gap junction synchrony. In contrast, the Downbound module retains a wider range of IO firing rates. The revised text (lines 681-687) now reflects this distinction more accurately.*

- l. 461: To my understanding, the authors studied a model in two different parameter regimes that are meant to correspond to upbound and downbound micromodules. In the absence of any interaction or integration of both in a larger network, the formulation of a cerebellar model comprising both modules is misleading.

*We thank the reviewer for this comment. We now realize that the original phrasing could be misinterpreted to imply that both Upbound and Downbound micromodules were simulated as interacting components within a single network model, while in fact, we studied two distinct parameter regimes representing these modules independently. The goal was to compare their emergent dynamics under matched input conditions, rather than to model their joint operation within a larger integrated cerebellar architecture.*

*We have revised the relevant paragraph to clarify this point and we hope to now avoid the implication that we studied a unified, interacting network model (lines 562- 569).*

- The title should reflect that this study investigates a model and doesn’t report experimental findings on the cerebellum (e.g. “A model of homeostatic bidirectional plasticity in Upbound and Downbound micromodules of the olivocerebellar system”).

*We agree that the title should clearly indicate the modeling nature of the study. Accordingly, we have revised the title to explicitly reflect the computational approach and focus on modular plasticity within the olivocerebellar system. The new title reads: “Homeostatic bidirectional plasticity in Upbound and Downbound micromodules in a model of the olivocerebellar system”*

The methods are partly sloppily written. Please fix:

*We thank the reviewer for this thorough and constructive feedback on the Methods section. We have carefully revised the text to address each point and improve clarity, consistency, and precision throughout.*

- l. 602: Gaussian white noise

*Corrected.*

- Table 1: units of \sigma_OU

*Corrected.*

- l. 622: Sampling of parameter values is unclear: uniform? evenly spaced?

*We have now clarified the sampling procedure in the Methods (lines 783-787). For each parameter we generated a population-sized list of values evenly spaced across the specified range. These values were then assigned without replacement to the cells, ensuring a uniform coverage of the parameter space across the population.*

- Eq. numbers are shifted / don’t correspond

*Corrected.*

- l. 644: firing → current

*Corrected.*

- Some formulations are misleading: l. 645 “current is 0 nA when there is no spike” although it is a filtered version of the spike train and will hardly be 0 ever.

*Corrected.*

- the nomenclature changes at some places (confusing subsubscripts)

*Corrected.*

- l. 668: The reference to the eyeblink experiment is unexpected - if description of simulation underlying Fig. 3 please be much more explicit on how those were done.

*Corrected.*

- Tables 2, 4: Several parameter variations are very small and can be expected to be irrelevant, while others are much more important, whereas no variations at all are considered for the values given in Table 6. Please comment.

*The variation ranges in Tables 2 and 4 were chosen to reflect known biological heterogeneity (PC and IO firing rates, from Zhou et al., 2014 and Voerman et al., 2022). We agree that in some cases the variation is small and may have limited influence on global dynamics; these were included for completeness and transparency.*

*Regarding Table 6, the parameters listed there define synaptic strengths and scaling constants that were kept fixed across simulations to isolate the effects of plasticity and modular organization. Varying these values would introduce additional degrees of freedom, complicating the interpretation of the main results.*

*We now explicitly state in the Methods (lines 873-881) our choices for parameters.*

- Presentation of BCM rule sloppy (what is “our BCM”?); the main equations are not the ones used, dimensions are inconsistent (Eq. 16,17) (have only currents for PF a priori, no def of \rho_{PF}, \rho_{PC} given) ; l 739 minimum wrong.

*We thank the reviewer for this thorough and constructive comment. We have revised the presentation of the BCM rule in the Methods section to provide a clearer and more consistent formulation, including all variables, dimensions, and functional dependencies. Specifically:*

- *We now explicitly define the PF and PC activity terms as firing rates (in Hz) denoted \rho_{PF} and \rho_{PC}, rather than using ambiguous current-based terms.*
- *We clarify that \rho_{PC} refers to a moving average over a 200ms window, and that the sliding threshold also evolves dynamically according to this average.*
- *Parabola minimum corrected.*
- *All equations have been checked for dimensional consistency.*
- *We no longer refer to the formulation as “our BCM” but describe it explicitly as a modified BCM rule with a homeostatic threshold and tanh damping function.*

*These changes are now reflected in the Methods section (lines 899-906).*

- l. 760: “instantaneously” contradicts long synaptic delay

*We thank the reviewer for this clarification. While the word “instantaneously” was intended to describe the implementation of the PC-CN synapse as a discrete-time event in the simulation, we agree that it may be misleading in the context of biological transmission. We have revised the wording (lines 938-940) to reflect that the synaptic current is updated immediately upon the presynaptic spike, without implying zero transmission delay in a biological sense.*

- l. 772: release of GABA into the cell?!

*Corrected.*

- Eq 26: V measured in which units?

*We thank the reviewer for pointing this out. In Eq. 26, v refers to the transjunctional voltage between two electrically coupled IO dendrites defined as* $V=V_{d}-V_{de}$ *where both voltages are expressed in millivolts (mV).*

- ll 798/99 unclear

*The intent was to describe how IO-to-PC connectivity was implemented while preserving convergence constraints. We have revised the text for clarity. Specifically, each PC is either assigned a unique IO input or connected to one selected from a predefined pool of IO neurons, without replacement, depending on the target convergence ratio (lines 977-981).*

- More generally, the two-step presentation of the model is somehow misleading, with first a definition of a baseline model (Table 6) and then different parameter values given in Table 7.

*We agree that the relationship between Tables 6 and 7 needed clearer explanation. Table 6 presents the fixed baseline synaptic parameters used in all simulations unless specifically overridden. Table 7 lists the parameters that were selectively modified across micromodules (Upbound vs. Downbound) and coupling conditions (Coupled vs. Uncoupled). These values replace their counterparts in Tables 4 and 6 when applicable, allowing us to simulate zone-specific dynamics and plasticity effects while maintaining a consistent core model. We have now revised the captions and Methods text to clarify this relationship (lines 990-994).*

- l 827/28 unclear: What was done to the weights, and why?

*We have revised the text to clarify the procedure (lines 1011-1018). Specifically, during each plasticity epoch, the PF-PC synaptic weights were allowed to evolve under the influence of the BCM and LTD rules in response to a fixed “frozen” PF input. At the end of each epoch, we smoothed the weight trajectory using a Savitzky-Golay filter to reduce the noise and extract a stable weight estimate. The final value of this filtered weight trace was then used as the fixed synaptic weight in the subsequent “after plasticity” condition. This approach allowed us to asses the network dynamics under stable, learned weights while maintaining consistent input conditions across epochs.*

**Reviewer #2:**

6-Please justify the chosen ratio of PCs, CN and IO cells.

*We thank the reviewer for this point of attention. The chosen ratios of PCs, CN and IO neurons in our model reflect known anatomical convergence and divergence patterns within the olivocerebellar system. Specifically, anatomical studies from mice (note that there is considerable variation across species) suggest that ~20-30 PCs converge onto a single CN neuron, while each PC projects to ~30-50 CN neurons [1]. Each IO projects to ~7-10 PCs, while each PC receives from a single CF [2,3].*

*To maintain biophysical plausibility while keeping the model computationally tractable, we modeled 100 PCs, 40 inhibitory CN neurons and 40 IO neurons per micromodule. This ratio captures the convergence from PCs to CNs and the broad divergence from each PC. We focus only on inhibitory CN neurons as they form the output of the cerebellar cortex and project to the IO [4–8]. For the CN-IO loop, we assume a 25% reciprocal connection probability: each CN neuron projects to ~10 IO neurons and each IO neuron receives from ~10 CN neurons. The design choices are now clarified in the Methods section in a new subsection titled Network architecture (lines 731-742).*

7-Fig. 2: please specify units for the synaptic weights.

*In our model, synaptic weights are unitless and represent normalized efficacy values used in a phenomenological learning rule. We have now clarified this in the caption of Fig. 2.*

8-Fig. 3A: no vertical scale is shown for the experimental recordings. Please explain the difference in timescale with the model results shown in B.

*We clarify that Fig. 3A is not a raw experimental recording but a sketch from a review article, intended to illustrate typical PC activity patterns across zones. As such, it does not include a vertical scale. To avoid confusion, we now explicitly state this in the figure caption. Regarding the difference in timescale between panels A and B, we have added an explanation in the caption noting that the model output in B is of a longer simulation window.*

9-Please justify the different statistical tests used to present the model results throughout the paper.

*We used two different statistical tests depending on the characteristics of the data being compared:*

- *Two-sample Student’s t-test were used when comparing means between two independent groups with approximately normal distributions (e.g. SSpk or CSpk firing rates across Upbound and Downbound zones).*
- *F-tests (using the complementary cumulative distribution function of the F-distribution) were employed to assess differences in variance between groups (e.g. variability of the CF pause duration or spike rates across plasticity conditions).*

*All statistical comparisons were two-sided and group sizes (number of cells) are indicated in each figure caption. We have now added this explanation to the Methods section under a new subheading titled “Statistical Analysis” for transparency and reproducibility (lines 1030-1036).*

10-For the reader it is not clear where the PSC is being measured in Fig. 4 (by the way the acronym is not defined until much later). In general in Fig. 4 and throughout the paper, it is not clear what experimental results require fine tuning to be reproduced.

*We have now clarified the meaning of the acronym PSC in the caption of Fig. 4 and in the text (lines 386-388). We specify where it is being measured —namely, entering the soma of the PC. Additionally we now explicitly state in the main text which experimental features required fine-tuning.*

11-Line 644: Eq5 -> Eq 6.

*Corrected.*

12-Line 820: Sampling rate is not an integration timestep. Please clarify this issue.

*We thank the reviewer for pointing out this seeming inconsistency in the methods. Indeed, the sampling rate of voltages, 1kHz, is different from the integration timestep of 0.025 ms. However, sampling rate here corresponds to the sampling rate of the filtering method, i.e. the sampling rate of the frozen noise, which is equal to the inverse of the integration timestep, as we sample the noise for each timestep. To make this distinction more clear, we have slightly adapted the text (line 999) to read:*

*[...] taking into account the rate at which the noise was sampled as (fs=1/dt) [...]*

13-Please indicate how many spikes were used for the analysis shown in Fig. S3.

*We thank the reviewer for pointing this out. We have clarified the sample size used for each analysis by using the number of cells (n) involved in each comparison in the caption of Fig. S3. We have done this for the statistics of the rest of the figures when pertinent and in a new section in the Methods called Satistical Analysis (lines 1030-1036).*

14-Please add the code GitHub link in the text.

*We thank the reviewer for this suggestion. We have now added the GitHub repository link for the model code to the main text. The following sentence has been added to a new subsection called Code (lines 1027-1029):*

*“All code used for simulations and analysis is available at:* [*https://github.com/eliasmateo95/CerebellarLoop*](https://github.com/eliasmateo95/CerebellarLoop)*”*

**References**

1.	Cerebellar dentate nucleus: Organization, cytology and transmitters, by Victoria Chan‐Palay, 550 pp, illustrated, $98.00, Springer, Berlin and New York, 1977. Ann Neurol. 1978;4: 588–588.

2.	Schmolesky MT, Weber JT, De Zeeuw CI, Hansel C. The making of a complex spike: ionic composition and plasticity. Ann N Y Acad Sci. 2002;978: 359–390.

3.	Busch SE, Hansel C. Climbing fiber multi-innervation of mouse Purkinje dendrites with arborization common to human. Science. 2023;381: 420–427.

4.	Ruigrok TJ. Cerebellar nuclei: the olivary connection. Prog Brain Res. 1997;114: 167–192.

5.	Scheibel ME, Scheibel AB. The inferior olive; a Golgi study. J Comp Neurol. 1955;102: 77–131.

6.	Vrieler N, Loyola S, Yarden-Rabinowitz Y, Hoogendorp J, Medvedev N, Hoogland TM, et al. Variability and directionality of inferior olive neuron dendrites revealed by detailed 3D characterization of an extensive morphological library. Brain Struct Funct. 2019;224: 1677–1695.

7.	Scheibel ME, Scheibel AB. Neuropil organization in the superior olive of the cat. Exp Neurol. 1974;43: 339–348.

8.	Brodal A, Scheibel A, Scheibel M, Walberg F. Areal distribution of axonal and dendritic patterns in inferior olive. J Comp Neurol. 1956;106: 21–49.
