# Supplementary material for: Homeostatic bidirectional plasticity in upbound and downbound micromodules in a model of the olivocerebellar loop
Source: PLoS Comput Biol. 2025 Oct 21;21(10):e1013609. doi: 10.1371/journal.pcbi.1013609 (PMC12571319; doi:10.1371/journal.pcbi.1013609)
Supplement: Attachment — Submitted filename: Response_to_Reviewers_auresp_2.docx [file pcbi.1013609.s013.docx]

***We thank the reviewers for the careful attention they gave to our manuscript and constructive feedback. We have edited the text throughout to address reviewers’ comments.***

***Our point­-by-­point response to comments is given below.***

**Reviewer's Responses to Questions**

**Reviewer #1**: While the overall clarity of the manuscript has indeed been improved, my main questions/difficulties with the study have not been satisfactorily addressed.

*The reviewer’s critical questions and points were instrumental in improving our delivery. We deeply appreciate the attention and work you devoted to reviewing our manuscript once more, and we hope that you will find this current version acceptable.*

**Reviewer #2:** The authors have addressed all my concerns raised in the first review iteration. The revised manuscript is significantly improved and provides clearer explanations of the model assumptions, derived results, and limitations. Units are missing in table 7. Otherwise, the paper is now ready for publication. I enjoyed reading this latest version of the manuscript. Congratulations on your work

*Thank you, we sincerely appreciate the positive feedback. The units missing in table 7 have been added.*

**Main points**

**Reviewer #1:**

1. “Baseline homeostasis” of up- and downbound modules in the presence of ongoing activity vs. procedural learning - the manuscript remains ambiguous which of these two effects are actually addressed, and remains short of an answer of how both experimental observations might be reconciled.

*We agree that the manuscript would benefit from a greater clarity on the distinction between baseline homeostasis and procedural learning. Our model was explicitly designed to study how ongoing plasticity can unify procedural and homeostatic learning, hence the repeated frozen noise representing contingencies of sensorimotor input. Our model yields stable synaptic configurations under fixed, ongoing input conditions. While the scenario addressed is more closely aligned with baseline homeostasis than with procedural learning, the implications of our findings bear on both. We now clarify this in both the introduction (lines 142-155) and discussion (lines 777-781), and explicitly state that while our model captures stabilization mechanisms under continuous input, it does not simulate specific learning-related changes driven by structured task contingencies. We also note that reconciling both processes, baseline homeostasis and learning-related adaptation, remains an important direction for future work.*

2. How does increased CN input increases IO output / CF rate? The authors propose ‘likely’ causes without providing evidence in that sense, and refer to experimental literature that has not shown such an effect. On the contrary, the optogenetic study by Chaumont et al. suggests that decreased CN activity (by stimulation of upstream PCs) leads to increased IO discharge.

*We appreciate this important clarification. In the revised text (lines 457-471), we rephrased our interpretation to avoid any suggestion of a direct excitatory drive from CN to IO. Instead, we state that “CN activity modulates IO excitability indirectly, through effects on STO phase and amplitude". This revision aligns our explanation with the findings of Chaumont et al., highlighting the role of inhibitory modulation of IO oscillatory dynamics rather than suggesting a direct drive of IO spiking by CN input.*

3. The authors do not test how their model responds to repeated exposures to new instantiations of OU input. A comparison between such a scenario and theirs remains crucial to assess what part of the observed stabilisation is due to the repeated temporal contingencies in the input (eventually relatable to learning) and what part is due to continuously fluctuating inputs (addressing the issue of weight homeostasis).

*We thank the reviewer for this comment. In the original simulations (Fig. 2G-L), we used a single frozen instantiation of OU noise to evaluate the system's capacity for synaptic stabilization under stationary but temporally correlated input. To assess the robustness of these findings, we now ran 10 additional simulations), each with independently generated OU inputs and randomly initialized networks (as described in the Methods, where intrinsic cell parameters are randomized per seed). For each simulation we computed the median absolute weight change between successive plasticity epochs, replicating the analysis from the old Fig. 2K-L (current Fig. 2I-J) but summarizing the results across simulation seeds rather than across individual synapses.*

*The results (new Fig. 2K-L) show consistent trends across input and network realizations: Upbound modules exhibited initial weight potentiation followed by stabilization around a new mean baseline (with only minor fluctuations), while Downbound modules showed early weight depression followed by similar stabilization. These results confirm that the bidirectional weight evolution observed in the original simulations is not an artifact of specific input structure or network initialization, but rather reflects intrinsic properties of the model dynamics under continuously fluctuating, frequency-rich input. We have added this analysis as part of figure 2 (panels M and N) and refer to it in the main text (lines 311-318).*

4. The authors report in their reply that “expected biological behavior” of firing rate changes is only observed for a “narrow range” of threshold timescale values. This suggests the model has been fine-tuned at least to some degree. The range of values should be reported, the value related to known plasticity mechanisms and the discussion not hidden in the methods (btw. on l. 908-917 and not where stated in the reply).

*We appreciate the reviewer's concern and welcome the opportunity to clarify. While our model exhibits stable weight dynamics, this is not the result of fine-tuning the BCM plasticity rule. Instead, we selected parameters based on biological constraints known from experimental literature. Specifically, the threshold time constant was chosen in the range of 10-20ms, corresponding to the typical duration of CF-triggered pauses in PC firing. We also set the maximum firing rate for potentiation to 250Hz, to exclude non-physiological PC activity.*

*Crucially, these values were selected a priori on biological plausibility, not to produce specific plasticity outcomes. Only after running the simulations did we observe that plasticity directions aligned with experimental findings (net depression in Downbound modules, potentiation in Upbound). We have now added this clarification to the Results section (lines 274-279) to make our rationale transparent. We also explicitly report the tested range (10-20ms), over which the BCM rule consistently yielded stable convergence with biologically plausible firing rates and directional changes in plasticity.*

5. The model description is somewhat improved, but several points raised in the first review have not or only insufficiently been addressed.

*All points have now been addressed.*

**General comments**

**Reviewer #1:**

- l. 85: “In this model” refers to which model? Connection not clear.

*Corrected*

- l. 87-88, l. 109-119: Resumes perfectly well the issue I have with the concept of homeostasis presented here. The long-term adaptation to input statistics should be the background on which actual learning operates. The feedback loop is studied without any coupling to motor errors or any other output-related, environment-mediated CF feedback; only intrinsic activities are considered. Under the hypothesis that plasticity is continuously operating and not switched on on a case-by-case basis, the feedback loop should indeed set a stable, “homeostatic” baseline state. In this setting, I would expect upbound and downbound modules to be the resulting baseline states as they are observed experimentally, not simply initial conditions before plasticity operates. Whether those resulting baseline states then further behave as observed experimentally during learning (with some form of learning-related, specific CF feedback), is another question. A true insight would be how these two experimental observations (stable, homeostatic modules, with corresponding biased firing rate modulations upon learning) can be reconciled.
*We agree that our model primarily captures baseline homeostasis in the absence of explicit motor error-related CF feedback. To address this point, we now explicitly clarify in the Introduction (lines 142-155) that the stable Upbound and Downbound states we observe emerge from ongoing loop dynamics and represent a homeostatic baseline.*

*Additionally, we have extended our results with a targeted simulation of a classical eyeblink conditioning paradigm (new Fig 7, S6-1 and S6-2). This simulation tests how stable Upbound and Downbound states established by ongoing plasticity respond when task-related CF inputs are introduced. The results demonstrate that our model can reproduce key aspects of bidirectional plasticity and firing rate modulations seen in experimental learning paradigms, thereby bridging baseline homeostasis with learning-related plasticity.*

*We discuss these new findings in the revised Introduction (lines 151-155), Results (lines 602-663), Discussion (lines 812-820) and Methods (lines 1141-1146, 1169-1173), which explicitly address how baseline homeostatic states may form the scaffold upon which supervised CF-drive plasticity operates.*

- l. 120-146: Somewhat in contradiction to what precedes, the authors relate their model here more directly to procedural learning, suggesting that they study the actual process of learning rather than homeostasis during ongoing activity. This apparent inconsistency needs to be clearly resolved. More generally, it seems that the authors do not (potentially cannot) address how both types of plastic changes (ongoing & homeostatic, leading to stable baseline states on the one hand, and learning-related, leading to modified weight distributions and firing rates on the other hand) can coexist.

*Our intent was to examine how ongoing plasticity under reverberating loop dynamics can give rise to stable, interpretable configurations and thereby modeling the homeostatic regime of the olivocerebellar system, rather than learning per se. However, we recognize that certain phrasing suggested that the model simulated task-driven adaptation. We have revised this section of the introduction (lines 142-154) to clearly state that our results reflect a baseline, pre-motorlearning state in which the loop dynamics drive synaptic organization in the absence of behaviorally contingent error feedback.*

- l. 165-167: “test repeated exposures to the same input” and especially l. 171-171 “a crucial aspect of our model is that we repeated presentations” - In its current form, the article does not allow the reader to appreciate whether the results depend on stimulus repetition or not; this should be clearly stated and shown.
*We apologize for the confusion caused by our phrasing. To clarify: our model does not include stimulus repetition in the experimental sense. Rather, we use a single instance of temporally structured Ornstein-Uhlenbeck (OU) input (what we referred to as “frozen input”) which is held constant across all simulations. This fixed input stream is not meant to mimic repeated stimulus events, but serves as a controlled input. It allows us to isolate the effects of plasticity by ensuring that all observed differences across conditions (e.g., before vs. after plasticity) arise solely from changes in the internal network dynamics, not from changes in the input. We have revised the manuscript (lines 182-185 and 188-193) to avoid the misleading terms “repetition” or “repeated exposure” and to clearly state that the input is constant throughout all experiments.*

- Fig. 2A: It is still not clear how to read this plot. What does the y-axis represent? What is represented by the gray-value shading? The color map doesn’t have a label, please also provide a unit for numerical values.
*We thank the reviewer for pointing this out. We have updated the caption of Fig. 2A to clarify the meaning of the y-axis, the role of the gray curves and the interpretation of the colormap. Specifically, the y-axis is* $\phi_{i}$ *which represents the general change in plasticity of the i-th PC which is then modulated by* $\rho_{PF_{j}}$ *for each PF bundle* $\phi_{i}=\frac{\rho_{PC_{i}}\left( \rho_{PC_{i}}-\theta_{M_{i}} \right)}{\theta_{M_{i}}}$*. In other words, it represents the magnitude of the BCM-driven synaptic change prior to scaling by PF activity. Each gray curve shows the BCM function for a different value of* $\theta_{M}$*, which is also encoded by the colormap (*$\theta$ *in Hz). We now explicitly label the colormap and include the units in the revised figure. Furthermore, we corrected the values in the axis of the colormap.*

- Fig. 2D-F: Weight or weight update (in which units)? It is not clear why “CSpk weights” (what does this mean?) should decay to baseline. Also, please replace “theta” by the appropriate greek letter throughout the figure caption (as used in the MS and the figure itself).

*In the revised figure caption we now clarify that Panels D-F show synaptic weight changes, not absolute weights. Panel D shows the change due to the CSpk-triggered LTD component, Panel E shows the change from the BCM component, and Panel F shows the total update per time step as the sum of both components. The units are arbitrary (unitless) and reflect normalized model efficacy values. We now indicate this explicitly in the figure caption.*

*We understand that “CSpk weights” was an imprecise phrase –we now refer to the CSpk-triggered LTD component of synaptic plasticity instead. The “decay to baseline” in Panel D reflects the fact that this LTD component is only triggered by IO (CSpk) events and decays in between them (during which LTP occurs).*

*We have also replaced all instances of “theta” in the figure caption with the correct Greek symbol, consistent with its appearance in the figure and text.*

- l. 317: Not clear from Fig. S2-1A how the global phase and “delta” phase are determined.

*We have revised the text (lines 363-366) to better explain how the global and delta phases were determined. Specifically, we now clarify that the global phase was calculated as a mean Hilbert phase of all IO cells, representing the overall phase progression of the network. The delta phase was defined as the difference between the Hilbert phase of each individual IO neuron and the global phase at each time point. This makes it clear how both measures were derived and how they are interpreted in Fig. S2-1A.*

- l. 350: “these results” is not clear. What results is this referring to?

*We revised the text (lines 402-404) to clarify that the results refer to the direction of synaptic plasticity: potentiation in Upbound and depression in Downbound.*

- Fig. 3A,B: This comparison still seems meaningless/misleading to me. The authors now seem to suggest so themselves in the main text (l. 339-349), which makes it even harder to understand why they kept this figure. If I would try to follow their (possible) reasoning and make sense of this comparison, then perhaps the timecourse of the SSpk rates AFTER stimulus onset over the course of learning, as indicated by the white arrows, might correspond to the (slow) changes in the SSpk firing rate during plasticity. This cannot be appreciated by this plot (besides the more general issue of learning vs. spontaneous weight updates).
*We understand the concern regarding Fig. 3A,B. The intention was not to equate the timescales or trial-locked dynamics between experiments and the model, but rather to illustrate that the directionality of SSpk modulation (potentiation vs. depression) observed experimentally is also captured by our model, albeit over much slower timescales. To clarify this, we have (1) revised the text (lines 394-404) to explicitly state that Fig. 3A is a schematic summary and not a direct experimental trace, (2) reworded the legend and main text to emphasize the difference in timescales and experimental conditions, and (3) added a sentence in the main text explaining that the relevant comparison is the direction of the change, not the precise time course.*

- Fig. 3B: What kind of averaging or smoothing do the thick lines represent in the model part? It is unclear how the firing rates can decrease before the onset of plasticity.

*In Fig. 3B, the thin translucent line is the raw (unsmoothed) population-averaged spike rate (1-s bins) obtained by averaging across PCs. The thick line is a Svitzky-Golay smoothed version of the same trace, applied across the entire concatenated sequence of the three epochs. Because the smoother spans across epoch boundaries, small boundary effects can appear at transitions (vertical dashed lines). This explains the apparent decrease just before the onset of plasticity despite no change in the raw (unsmoothed) trace. We have clarified this in the figure legend and Results (lines 394-404).*

- l. 372: “indemnified” → identified

*Corrected*

- Fig. 3C-E: It is not clear how model-cells of both modules (parameter sets) relate to each other, as suggested by the gray lines (no pre-post paradigm, treatment conditions etc.). Please explain or remove lines.
*We thank you for pointing this out. The grey lines indicate the pairwise comparisons between individual cells from the Upbound and Downbound modules. Each line connects a single cell in one module to its corresponding cell in the other module. These pairs share identical parameters set, except for PCs, the* $g_{Cal}$ *and* ${I^{(IO)}}_{OU}$ *parameters of the IOs, as detailed in Table 7. We have now clarified this explicitly in the figure legend.*

- l. 390: table states 50 ms CN-IO synaptic delay vs. 60 ms here

*Corrected*

- l. 398-399: Not clear how Fig. S3E supports the statement.

*We thank the reviewer for pointing out that the supporting figure for the statement (S4E) lacked a sufficient explanatory caption. We have now revised the figure caption to clarify that this panel shows the relationship between CN inhibition and IO dynamics. Specifically, the figure demonstrates that increasing the number of CN inhibitory synapses onto IO neurons lowers the STO frequency and increases the rate of IO spikes. This supports the statement that stronger CN inhibition slows IO STOs, while increasing the opportunity for spiking. We have also revised the main text for clarity (lines 457-471).*

- l. 399-401: Repetition, and no evidence given.

*We have revised this section (lines 457-471) to eliminate repetition and rephrased the interpretation more cautiously. Rather than asserting a direct effect, we now describe how CN inhibition may influence IO excitability by modulating the phase and amplitude of STOs, creating conditions that increase or decrease the likelihood of spiking. This links CN activity to IO output without overstating causality.*

- l. 402-403: “how amplitude of CN-triggered IO responses were more sensitive to PF-PC plasticity.” unclear. More sensitive than what?

*We have clarified this sentence in the revised text (lines 457-471). Rather than using the term “more sensitive”, we now state that “IO responses to CN spikes varied depending on prior synaptic changes, showing that the same input could produce variable IO responses depending on network history”. This rephrasing makes it explicit that the modulation is relative to the plasticity state of the network, not to a different condition or group.*

- l. 403: “This variability in CSpk timing” → variability of CSpk timing hasn’t been mentioned before, so unclear what this refers to.

*We now explicitly introduce this concept in the revised text (lines 457-471), stating: “This is consistent with experimental observations of trial-to-trial variability in CSpk timing”.*

- l. 425: “CN inhibition” potentially misleading (I misread as “inhibition of CN”), replace by “inhibitory CN input”?
*We agree that the original phrasing could be misleading and have revised the figure caption to read “inhibitory CN input", clarifying that it refers to the output from the CN targeting the IO.*

- l. 450-451: Which elements of the circuit (feedback loop) have been disregarded before?
*We agree that the phrasing was ambiguous. Our intention was to indicate a shift in focus: whereas the previous section emphasized local input features driving CSpk generation, here we broaden the scope to consider how those inputs propagate through and are shaped by the full olivocerebellar loop. We have rephrased this sentence accordingly to improve clarity (lines 516-518)*

- l. 498-499: Still not clear. If one assumes that the “spike opportunity” for a STO corresponds to the time spent above a given voltage threshold, then for a decreased frequency obviously the time above that threshold increases, but so does the time below threshold. The relative proportion of time, which to first order corresponds to the average excitability or “spiking opportunity” will remain the same.

*Indeed, this point is more involved than our description suggests. We are not referring to the windows of opportunity for spiking from the STO’s depolarization phase. To see how more hyperpolarization leads to more promptness to spike, we need to understand the channel dynamics of the IO model cells, which we have not emphasized in this work, though in other previous publications on the same IO cell model (Schweighofer et al., 1998, Warnaar et al., 2019, Loyola et al., 2023). Essentially, we refer to this spike opportunity as the tendency to spike when there is a pause in inhibition from DCN cells. Steady inhibition keeps the cells more hyperpolarized and dampens the oscillation amplitude. The result is that there is a sustained higher driving force with more frequent inhibition (as the membrane potential is farther from rest) and depolarizing channels, (particularly Calcium T-type), are more de-inactivated. This leads to more promptness to spike when there’s a release of inhibition, and the Calcium T-type drives a steeper depolarization slope. We have reviewed this description in the text and have cited the publications where these dynamics have been described.*

- l. 517,518: Please replace terminology “zebrin-positive/negative” in line with response to comment in round 1.

*Corrected, we have replaced zebrin- positive/negative with Upbound/Downbound.*

- l. 550, 553, 586, probably other instances: “continual” → continuous

*Corrected.*

- l. 560-561: With the formulation “intrinsic learning”, it is again not clear whether learning or homeostasis in the presence of ongoing plasticity is addressed.

*We have revised the sentence (line 677) to clarify our intended meaning. Specifically, the model focuses on homeostatic stabilization mechanisms arising from loop-intrinsic dynamics rather than learning per se.*

- l. 585: “weights that oscillated” is still not clear. Oscillations imply that there exists a period of that oscillation. Please characterize the strength of the weight fluctuations across epochs (ideally as a function of epoch length, and for repeated/frozen vs. newly drawn patterns, see also previous comment to l. 165-167).

*To clarify, we did not mean oscillations in the strict periodic sense, but rather small fluctuations in synaptic weights around a stable mean. These fluctuations reflect the ongoing balance between BCM potentiation and CSpk–triggered LTD under stationary input. We now rephrased the manuscript (line 702) to avoid the term “oscillation” and instead describe the weight dynamics as residual fluctuations.*

- l. 595: “underdamped” suggests indeed an underlying oscillatory dynamical system, which is not really shown here, see again earlier comments.

*We understand that the use of the term underdamped may misleadingly suggest a mathematically oscillatory system. To avoid this confusion, we have revised the text (lines 712-714) to use a more precise language that reflects the observed differences in convergence behavior between modules, without implying an oscillatory regime. Specifically, we now describe the Downbound modules as exhibiting slower convergence and greater residual variability, compared to the more stable convergence in Upbound modules.*

- The methods have improved, but the model description remains sloppy

*We have carefully revised the Methods section to address each point, improving clarity, and consistency throughout.*

- l. 765-766: How can it be that superscript i = PC, CN when the sum is carried out over i and cell-type specific currents?

*Thank you for this clarification request. We have revised the sentence (lines 902-903) to clarify that the index “i” refers to the different current components entering a given cell type (i.e., PC or CN), not that “i” loops over both PC and CN simultaneously.*

- l. 819: “differential equation {I_{IO}}_{CN}” What does this refer to? Also, please make sure to keep notations consistent: I^{(OU)}_{XY} is not the same as {I_{OU}}_{XY} (superscript with parenthesis vs. subscript without).

*Thank you for pointing this out. We have corrected the notation for consistency: the current is now denoted* ${I^{(IO)}}_{CN}$*, in line with the other currents used in the model. This current represents the inhibitory input from the CN onto the IO, and the corresponding differential equation is explicitly described in the Synapses subsection of the Methods. We have also revised the sentence for clarity (lines 953-954).*

- l. 876: symbol missing/wrongly represented

*Corrected.*

- l. 893: Why is \phi indexed by i?

*We indexed* $\phi$*by* $i$ *because each PC has its own firing rate* $\rho_{PC_{i}}$ *and sliding threshold, leading to a cell-specific plasticity response. Thus* $\phi_{i}$ *reflects the general change in plasticity for the* $i$*-th PC based on its individual activity history. We have clarified this in the text (lines 1033-1035).*

- l. 888: What is the instantaneous firing rate of the PF? Their activity has been described as an OU process representing current input, not fluctuating firing rates.

*While the PF input is generated as an OU process representing fluctuating synaptic current, we compute an effective activity measure, by converting the instantaneous input into an equivalent firing rate in Hz. This abstraction is used in the plasticity rule to enable multiplicative modulation of weight changes and aligns with previous rate-based BCM implementations. To clarify this, we have rephrased the sentence (line 1063-1066).*

- l. 898: “We need to damp this” - why? Formulation sounds funny for a methods section. Please make very explicit which parts of the BCM rule have been introduced earlier (relate to the original model or variants) and which parts / modeling choices are introduced by the authors.
*We have revised the Methods section for clarity and precision. We now explicitly state which parts of the BCM rule follow the classical formulation (Eqs 15-17) and which part is our modification (Eq 18), namely the hyperbolic damping term added to limit potentiation at high PC firing rates. We also removed the informal wording “we need to damp this” and replaced it with a clear rational that the damping ensures physiologically plausible weight updates at high firing rates (line 1037-1044)*

- Eq. 18: Doesn’t make sense, as the argument of tanh has the dimension of Hz. Please correct.
*Our implementation in Brian2 ensures the tanh argument is dimensionless (we explicitly non-dimensionalize the rate term). To avoid confusion we have clarified this in the Methods (line 1041-1044) that in the implementation in Brian 2 the argument is rendered dimensionless by multiplying by a time unit.*

- l. 904: Comparison with 0 doesn’t make sense as written (should be difference).
*We corrected the sentence to state explicitly that potentiation occurs when* $\rho_{PC}<\theta_{M}$ and depression when $\rho_{PC}>\theta_{M}$.

- Eq. 20: Dimension mismatch between \rho_{PF} and I_{PF}. Also, why is the change relative to deviation from mean PF input, with equally strong weight changes for stronger and lower PC activity?
*We have clarified in the revised text (line 1063-1073) that in Brian2, units are handled explicitly, and we include the appropriate scaling so that the term has units of Hz. Biologically, the purpose of Eq 20 is to measure the instantaneous deviation of the PF input from its mean level which is treated as a baseline constant. A larger deviation from baseline corresponds to a larger LTD update.*

*It is not the case that stronger and lower PC activities produce equally strong weight changes: PC activity is directly driven by PF input, so when PF input is high and deviates substantially from the mean, the resulting PC activity is high and weight changes are correspondingly larger. This formulation allows LTD to scale with the magnitude of PF-driven excitation.*

- More generally regarding the LTD weight changes: If w_CSpk represents a weight and not a weight change (question asked in first review, see also points above), then this rule also incorporates (aspects of) LTP because of the decay of the w_CSpk component (which correspond to positive weight changes). Please give a very decent thought to the definition and description of the plasticity rules.

*We clarified that the LTD term is an auxiliary variable representing a transient contribution to the synaptic weight change, not the synaptic weight itself. This variable always remains negative and decays back toward zero between CSpks. As it decays, it allows weight potentiation via the BCM term (if w_BCM > w_CSpk). We have revised the text in the Methods to make this explicit (lines 1063-1073).*

- l. 999-1006: Why was the temporal filtering necessary, when only instantaneous snapshots of weights are compared? How would the results change when no filtering was used (and unfiltered weights used as inputs for the next epoch)?

*Temporal filtering was applied to avoid biasing the extracted final weight by the instantaneous state of the LTD mechanism. For example, if the simulation ended immediately after a CSpk, the last sample of the weight would be disproportionately depressed, which would not represent the average state of the synapse at the end of the epoch. The Savitzky-Golay filtering provides a smoothed estimate of the weight trajectory, from which we extract a stable final value. We have clarified this in the text (line 1157-1162)*
