# Supplementary material for: Homeostatic bidirectional plasticity in upbound and downbound micromodules in a model of the olivocerebellar loop
Source: PLoS Comput Biol. 2025 Oct 21;21(10):e1013609. doi: 10.1371/journal.pcbi.1013609 (PMC12571319; doi:10.1371/journal.pcbi.1013609)
Supplement: Attachment — Submitted filename: Response_to_Reviewers_auresp_3.docx [file pcbi.1013609.s014.docx]

**We thank the reviewer for the careful attention they gave to our manuscript and constructive feedback. We have edited the text throughout to address reviewers’ comments.**

**Our point­-by-­point response to comments is given below.**

**Reviewer's Responses to Questions**

**Reviewer #1**: I acknowledge the effort the authors put into trying to address my concerns. Although the manuscript still suffers from some ambiguity regarding homeostasis vs. learning pointed out in my previous reports, I do now consider the manuscript in principle fit for publication as important clarifications have been made.

*We thank the reviewer for this encouraging evaluation and for recognizing the improvements made to the manuscript. We appreciate the opportunity to respond to the remaining points of ambiguity and to provide clarification.*

**Main points**

**Reviewer #1:**

1. Just in addition to my earlier reports, I would like to suggest the following thought experiment: Stick an electrode in a characteristic downbound or upbound zone and measure SSpk or CSpk rates in awake animals in the absence of any learning experiment being performed. What would be the measured baseline firing rates? Would these rates evolve over time? Would the authors argue that plasticity is operating or not during and in between recording sessions?

1. *What would be the measured baseline firing rates?*

*Defining the true baseline rate is an epistemic challenge. Clearly, SSpk and CSpk firing rates exist prior to learning. Baseline firing rates are observed and measurable in awake animals at rest, in the absence of any learning or conditioning protocol, but it is not obvious how to ascertain such an initial condition. The data presented in Figure 3, and the in-vivo recordings we reference from (Zhou et al. 2014), represent a presumed baseline, i.e., spontaneous activity in Up/Downbound PCs. These recordings were performed in awake animals not engaged in a behavioral task, and show stable differences in SSpk firing rates across zones.*

*Developmental evidence supports the view that zone-specific firing characteristics are not acquired through experience-dependent plasticity but are instead genetically programmed, via activity dependent mechanisms of ion channel expression. For example, (Beekhof et al. 2021) show that zebrin-based firing differences are already evident in juvenile mice before the cerebellar cortex is fully mature. Once the PC intrinsic properties (e.g. ion channel expression) are in place, zonal firing rate differences are robustly expressed. This supports the interpretation that the firing rate differences are not induced by learning but reflect stable, developmentally programmed features of Up/Downbound zones.*

2. *Would these rates evolve over time?*

*Over seconds to minutes, baseline rates appear remarkably stable, as shown in (Zhou et al. 2014) in their figure 2 (see below). While it remains an open experimental question how baseline activity evolves over the course of hours and days in the same cells, there is growing evidence that zone-specific firing characteristics are already present very early in development. On such longer timescales, firing rates may evolve gradually as part of development, particularly during early postnatal stages when PC morphology and ion channel expression are still maturing. During this window, PC firing rates are generally lower and more variable. Once the pacemaking machinery is fully expressed, however, the zonal firing rate differences become stable and persistent.*

3. *Would the authors argue that plasticity is operating or not during and in between recording sessions?*

*In alignment with the assumptions in our model plasticity is assumed to be ongoing and continuous. We model PF-PC plasticity as a combination of CSpk-dependent LTD and a homeostatic BCM-like mechanism. Although we artificially isolate “non-plasticity” periods in the model so as to compare “pre-” and “post-learning states”, we consider these as methodological tools, for clarity. Biologically, plasticity is always operating. This aligns with experimental observations showing that, even after learning, cerebellar activity returns to stable baseline firing patterns through ongoing plasticity (De Zeeuw 2021).*

*We now make these points explicit in the revised Discussion (lines 698-708).*

2. If the authors agree that firing rates remain stable and that plasticity is not gated during learning but operating continuously, this is the baseline regime that the authors should describe with their model *after* ongoing plasticity has lead to stationary weight distributions, and based on which they should distinguish up- and downbound modules according to their physiological criteria. In contrast, any hypothetical “initial”, before-plasticity network state should be considered experimentally inaccessible.

*We now clarify this distinction in the manuscript accordingly. We explicitly state that the after-plasticity condition corresponds to the physiological baseline state, shaped by ongoing loop dynamics under stationary input. In contrast, the before-plasticity rate is used as a modeling convenience for initialization, and while it is intended to represent a biologically plausible starting point, it does not reflect a stable or stationary network state. We revised the legend of Figure 3 and Methods (line 1174-1177) section to reflect this distinction. We should also acknowledge that, although our model only includes CSpk as a gating mechanism for plasticity, neuromodulators are still likely to affect time constants of plasticity, possibly in a gated way.”*

3. It may well be that I continue to miss something in the authors’ logic - but taken at face value, the final stationary SSpk rates of both modules that the authors report are actually inverted relative to experimental observations, i.e., the authors report higher baseline SSpk rates for the upbound module and lower baseline SSpk rates for the downbound module (Fig. 4C). In light of this result, the statement on l. 711 (“loop mechanisms enhance this functional baseline difference”) appears unwarranted if not wrong. Furthermore, it remains unclear, and I am obviously repeating myself, why the initial relaxation towards these baseline states is deemed comparable to any learning-related firing rate changes (as in Fig. 3A,B, notwithstanding the more clearly stated caveats), or more generally interpreted as plasticity-mediated stabilization of the modules’ baselines. The comparisons of Fig. 3, which are suggestive of a “match” between experiment and model, are misleading in that regard; to avoid any confusion, it should at least be stated in the legend of Fig. 3C-E that the model firing rates do not correspond to the homeostatic baseline state (after plasticity) but to the initial rates before plasticity is allowed to operate.

*We thank the reviewer for these observations. The firing rate differences between zones in our model indeed differ in magnitude (and direction) from some experimental reports. We have revised the text at line 711 to read: "However, the precise direction of baseline firing rate divergence between Up/Downbound modules may also depend on additional biological mechanisms not captured in our model, such as MLI inhibition or structured MF input”. Furthermore, we have updated the legend of Fig. 3 to clarify that the comparison is made to the direction of change, not to absolute firing rate values, and that the model panels (B-E) reflect simulated activity during plasticity, not yet a homeostatic equilibrium. The revised legend now reads: “Note that the model traces reflect activity during the plasticity epoch, before full stabilization, and are used to assess directionality of plasticity rather than absolute firing rates.”*

4. I appreciate that the authors now included additional simulations of actual learning on top of the baseline states obtained by ongoing plasticity. One may argue whether the implemented protocol, in which e.g. the strength of the US relayed to the IO does not depend on the PC activity, is a faithful implementation of cerebellar learning or eyeblink conditioning in particular, as increased eyelid closure should result in lesser stimulation of the IO. (Relatedly, it is not clear whether the authors considered this interaction in their comment ll. 612-614.) However, I understand that a more detailed simulation of eyeblink conditioning goes beyond the scope of this article. The data of Fig. 7F seem to suggest that the SSpk rates in up- and downbound zones do indeed change as expected during learning, which is a nice result. It would be helpful if the authors could state more clearly in the legend or methods how and when those rates were determined, as this cannot be deduced from Fig. 7E (where the effect is not clearly visible btw.).

*We have added a sentence to the Discussion (lines 856-858) explicitly acknowledging this limitation: “While the conditioning paradigm captures key elements of learning-related CF modulation, it does not implement full IO-gated feedback loops that modulate CF activity based on behavioral output.".*  *The SSpk rates reported in Fig. 7F were computed using the same method as for our other firing rate analyses throughout the manuscript (e.g. Fig. 3B, Fig. 4C, etc). Specifically, we calculated the mean firing rate over a 1-second window for the full simulation time, binned at 1-second resolution and averaged over the cell population. This includes all trials (US-only, CS+US, and CS-only), allowing for a robust estimate of the firing rate after plasticity. The statistical comparison shown in Fig. 7F reflects average firing rate differences across conditions and micromodules after learning. This method ensures consistency across all figures. We have now clarified this in a new subsection in the Methods (lines 1201-1207).*

5. One last point: On ll. 1014-1015 the authors now state that the effective PF firing rate is computed from the amplitude of the OU-driven synaptic current, but exactly how this calculation is done is not specified.

*We have now added the following explanation to the Methods (lines 1046-1051): "The effective PF firing rate,* $\rho_{PF}$*, is computed by transforming the instantaneous synaptic current (*$I_{PF}$*, in nA) into units of Hz by scaling with the amplitude of the OU process and assuming a linear mapping between current magnitude and presynaptic rate. This transformation is valid under the model assumption that PF input represents the activity of a large bundle of asynchronously active fibers whose summed conductance mimics a continuous input current.”*

**References**

Beekhof, Gerrit Cornelis, Catarina Osório, Joshua J. White, Scott van Zoomeren, Hannah van der Stok, Bilian Xiong, Ingo Hms Nettersheim, et al. 2021. “Differential Spatiotemporal Development of Purkinje Cell Populations and Cerebellum-Dependent Sensorimotor Behaviors.” *eLife* 10 (May). https://doi.org/[10.7554/eLife.63668](http://dx.doi.org/10.7554/eLife.63668).

De Zeeuw, Chris I. 2021. “Bidirectional Learning in Upbound and Downbound Microzones of the Cerebellum.” *Nature Reviews. Neuroscience* 22 (2): 92–110.

Zhou, Haibo, Zhanmin Lin, Kai Voges, Chiheng Ju, Zhenyu Gao, Laurens W. J. Bosman, Tom J. H. Ruigrok, Freek E. Hoebeek, Chris I. De Zeeuw, and Martijn Schonewille. 2014. “Cerebellar Modules Operate at Different Frequencies.” *eLife* 3 (May):e02536.
