# Supplementary material for: Homeostatic bidirectional plasticity in upbound and downbound micromodules in a model of the olivocerebellar loop
Source: PLoS Comput Biol. 2025 Oct 21;21(10):e1013609. doi: 10.1371/journal.pcbi.1013609 (PMC12571319; doi:10.1371/journal.pcbi.1013609)
Supplement: Attachment — Submitted filename: Response_to_Reviewers_auresp_4.docx [file pcbi.1013609.s015.docx]

**We thank the reviewer for the careful attention they gave to our manuscript and constructive feedback. We have edited the text throughout to address reviewers’ comments.**

**Our point­-by-­point response to comments is given below.**

**Reviewer's Responses to Questions**

**Reviewer #1:**

1. Please remove the sentence “loop mechanisms enhance this functional baseline difference”, which is not warranted by the simulation results, see my earlier reports. In their last reply, the authors indeed acknowledge that their results differ with experimental data in magnitude and direction (direction being the important part here, as the authors claim that the baseline difference is *enhanced* when their simulations show the opposite). In their reply, the authors seem to diminish the importance of this difference by referring to “some” experimental data, but the entire argument of their study is actually built on an observed baseline difference with strongly discharging downbound modules and lesser discharging upbound modules (see e.g. ll. 94-108).?

*Corrected. It now says: “the olivocerebellar loop mechanisms stabilize these differences through the interaction of BCM and CSpk-dependent long-term plasticity.”*

In ll. 600-601, the authors state that they will investigate how the previously identified baseline states evolve during a simulated learning paradigm. I apologize for not having paid more attention earlier, but it is not clear then how individual and mean firing rates of both modules as shown in Fig. 7E before CS onset and Fig. 7F left can be inverted relative to the homeostatic baseline firing rates reported in Figs. 4C & 5B (no filter). In Fig. 7, the baseline difference in density is ~0.45 (which units?) for the downbound module vs. ~0.3 for the upbound module, which amounts to a significant difference in baseline mean firing rates of approx. ~1.5 downbound/upbound, in marked contrast to roughly equal firing rates at baseline reported earlier (see also ll. 471-473).

*We thank the reviewer for their careful reading and for pointing out the apparent inconsistency between baseline firing rates in Figs 4C/5B and Figure 7E/F. We would like to clarify that Fig 7E shows firing rate densities across the population, whereas Figure 7F provides the actual distribution of mean firing rates across cells. In the “before plasticity” condition (Fig. 7F), Downbound PCs indeed fire at a higher average rate (~80 Hz) than Upbound PCs (~60 Hz), consistent with the same trend reported in Fig 4C. After plasticity, both populations converge toward ~65–70 Hz, again matching the results in Fig 4C. We have clarified this in the figure legend (now specifying that Fig 7E shows normalized firing rate density and does not reflect direct comparisons of population means).*

**Have the authors made all data and (if applicable) computational code underlying the findings in their manuscript fully available?**

The [PLOS Data policy](https://eur01.safelinks.protection.outlook.com/?url=https%3A%2F%2Ftrack.editorialmanager.com%2FCL0%2Fhttps%3A%252F%252Fjournals.plos.org%252Fploscompbiol%252Fs%252Fmaterials-and-software-sharing%2F1%2F010f0199be51cb48-eeb4cb15-53b8-4385-a7a9-d36e0414f214-000000%2F3kfGq_87NO9QOZad4OjyHlFK4lCK-ZoiGiPaQbTnBh4%3D231&data=05%7C02%7Ce.fernandezsantoro%40erasmusmc.nl%7Cd19ef7c1956543881c1108de059053a5%7C526638ba6af34b0fa532a1a511f4ac80%7C0%7C0%7C638954314632826607%7CUnknown%7CTWFpbGZsb3d8eyJFbXB0eU1hcGkiOnRydWUsIlYiOiIwLjAuMDAwMCIsIlAiOiJXaW4zMiIsIkFOIjoiTWFpbCIsIldUIjoyfQ%3D%3D%7C0%7C%7C%7C&sdata=thdM2b7sfqfxLaWLjet9EAUjRgUuLaVcE1FEOlUe37k%3D&reserved=0) requires authors to make all data and code underlying the findings described in their manuscript fully available without restriction, with rare exception (please refer to the Data Availability Statement in the manuscript PDF file). The data and code should be provided as part of the manuscript or its supporting information, or deposited to a public repository. For example, in addition to summary statistics, the data points behind means, medians and variance measures should be available. If there are restrictions on publicly sharing data or code —e.g. participant privacy or use of data from a third party—those must be specified.

Reviewer #1: None

*Yes. All code used for simulations and analysis is fully described in the “Code” subsection of the Methods (line 1205). It is available at*: <https://github.com/eliasmateo95/CerebellarLoop>.
